# Phylogenetic Analysis of Seven WRKY Genes across the Palm Subtribe Attaleinae (Arecaceae) Identifies *Syagrus* as Sister Group of the Coconut

**DOI:** 10.1371/journal.pone.0007353

**Published:** 2009-10-06

**Authors:** Alan W. Meerow, Larry Noblick, James W. Borrone, Thomas L. P. Couvreur, Margarita Mauro-Herrera, William J. Hahn, David N. Kuhn, Kyoko Nakamura, Nora H. Oleas, Raymond J. Schnell

**Affiliations:** 1 USDA-ARS-SHRS-National Germplasm Repository, Miami, Florida, United States of America; 2 Montgomery Botanical Center, Miami, Florida, United States of America; 3 Oklahoma State University, Department of Botany, Stillwater, Oklahoma, United States of America; 4 New York Botanical Garden, New York, New York, United States of America; 5 Georgetown University, Washington, DC, United States of America; 6 Florida International University, Department of Biological Sciences, Miami, Florida, United States of America; McGill University, Canada

## Abstract

**Background:**

The Cocoseae is one of 13 tribes of Arecaceae subfam. Arecoideae, and contains a number of palms with significant economic importance, including the monotypic and pantropical *Cocos nucifera* L., the coconut, the origins of which have been one of the “abominable mysteries” of palm systematics for decades. Previous studies with predominantly plastid genes weakly supported American ancestry for the coconut but ambiguous sister relationships. In this paper, we use multiple single copy nuclear loci to address the phylogeny of the Cocoseae subtribe Attaleinae, and resolve the closest extant relative of the coconut.

**Methodology/Principal Findings:**

We present the results of combined analysis of DNA sequences of seven WRKY transcription factor loci across 72 samples of Arecaceae tribe Cocoseae subtribe Attaleinae, representing all genera classified within the subtribe, and three outgroup taxa with maximum parsimony, maximum likelihood, and Bayesian approaches, producing highly congruent and well-resolved trees that robustly identify the genus *Syagrus* as sister to *Cocos* and resolve novel and well-supported relationships among the other genera of the Attaleinae. We also address incongruence among the gene trees with gene tree reconciliation analysis, and assign estimated ages to the nodes of our tree.

**Conclusions/Significance:**

This study represents the as yet most extensive phylogenetic analyses of Cocoseae subtribe Attaleinae. We present a well-resolved and supported phylogeny of the subtribe that robustly indicates a sister relationship between *Cocos* and *Syagrus*. This is not only of biogeographic interest, but will also open fruitful avenues of inquiry regarding evolution of functional genes useful for crop improvement. Establishment of two major clades of American Attaleinae occurred in the Oligocene (ca. 37 MYBP) in Eastern Brazil. The divergence of *Cocos* from *Syagrus* is estimated at 35 MYBP. The biogeographic and morphological congruence that we see for clades resolved in the Attaleinae suggests that WRKY loci are informative markers for investigating the phylogenetic relationships of the palm family.

## Introduction

*Cocos nucifera* L., the coconut, is a charismatic monotypic genus forming a dominant part of littoral vegetation across the tropics. Besides its paradisiacal connotation, the coconut plays a vital role at many different economic levels [1]. *Cocos nucifera* is pantropically distributed, a present day range significantly influenced both by a seed well-adapted to oceanic dispersal and the species' importance to humans [2]–[4]. Because of this wide geographic range, the biogeographic origins of the coconut have been one of the “abominable mysteries” of palm systematics for decades [5]. A neotropical origin of *Cocos* was first proposed by de Candolle [6]. Beccari [7] suggested an origin in Asia or the South Pacific, while Moore [8] proposed Melanesia. Harries [2] argued for a western Pacific origin, and later [3] opined for an origin in the Malesia biogeographic province (the Malay Peninsula, Indonesia, the Philippines and New Guinea), an opinion supposedly shared by a majority of coconut specialists [9], but weakly supported with data. Moreover, in the recent literature on the subject by coconut geneticists [3], [9], there does not appear to be a clear distinction between the deeper phylogenetic history of the genus and its far more recent domestication.

*Cocos nucifera* belongs to the monophyletic Cocoseae [5], [10]–[13], one of thirteen tribes of Arecaceae subfam. Arecoideae [14]. In addition to the coconut, this tribe also contains a number of other palms with significant economic importance, e.g., *Elaeis guineensis* Jacq. (African oil palm), *Bactris gasipaes* Kunth (peach palm), and many other species of value in local economies [15]. The tribe was first recognized informally by Moore [8] as “the cocosoid palms,” denoted by fruits with bony endocarps bearing three germination pores or “eyes.” He further delimited three subgroups as the *Bactris* Jacq., *Cocos* L., and *Elaeis* Jacq. alliances. Uhl and Dransfield [16] formalized the groups as tribe Cocoeae containing 22 genera classified within five subtribes, later reduced to 20 genera in three subtribes (more or less embracing Moore's [8] alliances), with orthographic correction of the name to Cocoseae [17]. In addition to the distinctive endocarp, the tribe is well-marked by its once-branched inflorescence, inconspicuous prophyll, conspicuous and often woody peduncular bract, imbricate petals of female flowers, and a triovulate gynoecium [14]. Cocoseae now encompasses 18–19 genera of predominantly Neotropical distribution [14]. One genus, *Elaeis* Jacq., has both a species endemic to tropical America and another in Africa.

Within the Cocoseae, the coconut is part of the moderate-sized subtribe Attaleinae [14], containing 11–12 genera. With the exception of *Cocos*, two endemics in Madagascar (*Beccariophoenix* Jum. & H. Perrier, two spp.; *Voanioala* J. Dransf., monotypic), and *Jubaeopsis* Becc. (monotypic, found in a restricted part of South Africa), the majority of the genera are Neotropical endemics [18].

Despite the importance of the Cocoseae, a well-resolved phylogeny for this tribe has been elusive [5], [10]–[13], [19]. Thus, to date, molecular systematics using plastid or nuclear markers have failed to unambiguously identify the sister genus of the coconut, determination of which is not only of biogeographic interest, but will also open fruitful avenues of inquiry regarding evolution of functional genes useful for crop improvement.

The utility of WRKY loci for determining infraspecific relationships has been demonstrated by genetic mapping in *Theobroma cacao* L. [20] and by differentiating individuals from one another within *T. cacao* [21] and *C. nucifera* germplasm collections [22], [23]. Borrone et al. [24] demonstrated the utility of WRKY genes for phylogenetic inference in Malvaceae. Further information on WRKY loci, including details of their evolution and orthology, can be found in the Discussion section of this paper.

Our study focuses on reconstructing the phylogenetic relationships within the subtribe Attaleinae, and represents the most intensive sampling of the group so far in a molecular analysis. We use sequences of seven putatively independent, single copy WRKY loci originally isolated from *Cocos nucifera* in order to resolve the closest extant relative of the coconut, the evolutionary relationships of the other genera in the subtribe, determine how paleohistorical events shaped the evolution and biogeography of the Attaleinae, and demonstrate the utility of WRKY loci for phylogenetic inference within the Arecaceae.

## Results

### Individual gene tree analyses

Excluding gaps, the number of phylogenetically informative characters (Table 1) ranged from 66 (WRKY12) to 271 (WRKY19). Maximum parsimony (MP) strict and maximum likelihood (ML) bootstrap consensus trees are available as supplemental Figures S1–4. Consistency indices (CI) were above 0.75 for all seven gene matrices investigated, while retention indices (RI) were always >0.89 (Table 1). Adding a coded indel matrix contributed little additional resolution to the trees, though bootstrap support (BP) was slightly increased for some clades (not shown). For each gene matrix, ML produced a tree identical in topology to one of the trees found by MP.

**Table 1:** Results of heuristic maximum parsimony phylogenetic analyses of seven WRKY loci across subtribe Attaleinae.

Locus	WRKY Group	# of trees†	Tree length	Total # of Characters	PIC‡	CI§	RI	Genera∥ (monotypic excluded) resolved as monophyletic in strict consensus (BP¥)	Suprageneric relationships from strict consensus (BP¥)
WRKY02	1	477	312	844	113	0.782	0.903	ALL (97), ATT (84), BUT (99), LYT (59), PAR (97)	ALL-POL (53), BUT-JUB (<50), ATT-BUT-JUB (50), LYT-SYA (95, LYT nested in SYA), BEC-JUBA-VOA (53), PAR-COC-(BEC-JUBA-VOA) (<50)
WRKY06	1	3	226	765	110	0.912	0.969	ALL (99), ATT (97), BUT (64), PAR (86)	ALL-PAR (72), ALL-PAR-POL (95), BUT-JUB (93), ATT-COC (<50), LYT-SYA (99, LYT paraphyletic with SYA), JUBA-VOA (66), (ALL-PAR-POL)-(BUT-JUB)-(SYA-LYT) (<50)
WRKY07	1	4286	383	829	165	0.804	0.897	ALL (100), BUT (84), LYT (73), PAR (99)	ALL-PAR (90), BUT-JUB (81), (BUT-JUB)-POL (89), BEC-JUBA-VON (89), COC-SYA “Amazonian” subclade (59)
WRKY12	1	263	154	769	66	0.844	0.925	ALL (67), ATT (82), BUT (<50), SYA inc. LYT (76), PAR (91)	ALL-POL (85), ATT-COC-SYA inc. LYT (52), BUT-JUB (96), (BUT-JUB)-PAR (51), JUB-VON (91), (JUB-VON)-BEC (only in bootstrap consensus, 61)
WRKY16	2c	786	294	658	110	0.756	0.890	ALL (99), BUT (100), LYT (99), PAR (99)	ALL-POL (<50), ATT-COC (55, ATT paraphyletic with COC)
WRKY19	2c	48	307	689	144	0.827	0.943	ATT (86), LYT (99), PARA (100), SYA (inc. LYT, 82)	ALL-POL (66), BUT-JUB (64), (SYA-LYT)-COC (76), ((SYA-LYT)-COC)-VOA (89)
WRKY21	2b	265	706	1277	271	0.781	0.901	ALL (90), ATT (100), BUT (100), LYT (100), PAR (100)	ALL-POL (95), BUT-JUB (100), JUBA-VOA (75)

* WRKY Group as originally defined by Eulgem et al. (2000).

† Branches collapsed if minimum length = 0.

‡ PIC = phylogenetically informative characters.

§ CI = consistency index.

¶ RI = retention index.

∥ ALL = *Allagoptera*, ATT = *Attalea*, BEC = *Beccariophoenix*, BUT = *Butia*, COC = *Cocos*, JUB = *Jubaea*, JUBA = *Jubaeopsis*, LYT = *Lytocaryum*, PAR = *Parajubaea*, POL = *Polyandrococos*, SYA = *Syagrus*, VOA = *Voanioala*.

¥ BP = bootstrap %.

#### Phylogenetic incongruence among the loci

Examination of the individual consensus trees and partitioned Bremer indices (Tables 1–2, Figs. S1, 2) indicates that incongruence is more a result of insufficient resolution at various nodes within each locus or “soft” incongruence, rather than conflicting resolution, or “hard” incongruence [25], though some degree of conflict at deeper nodes in the trees is evident (Figs. S1–4). P values from the incongruence length difference (ILD) tests [26], [27] indicated that the null hypothesis of congruence could be accepted for WRKY2 and 12, and 12 and 21 (p = 0.44 and 0.68, respectively). The latter are in different major clades of the WRKY family phylogeny (1 and 2b, respectively). P values for all other pairwise combinations were never lower than 0.01. The accuracy of the ILD as an arbiter of combinability has declined steadily since Farris et al. [26], [27] first recommended a P-value of 0.05 as the threshold for determining non-combinability. Numerous studies have concluded that P-values<0.05, and even as low as 0.001, should not preclude data set combination [28]–[36]. Based on these results, and the fact that many of the same monophyletic groups were resolved with each locus (Tables 1–2), we combined all seven loci for phylogenetic analysis.

**Table 2:** Partitioned Bremer (decay) indices (DI) and results of dispersal-vicariance *.

Node†	WRKY2	WRKY6	WRKY7	WRKY12	WRKY16	WRKY19	WRKY21	OPTIMIZED AREA**
1	−1.96	−0	−0.05	−0.01	1.07	2.97	−0.02	E
2	3.48	4	8.01	1.03	4.07	0.2	4.39	EF
3	−0.02	−1	−6.98	2.94	2.9	2.04	4.12	E
4	10.95	9	0.14	1.93	7.09	4.51	6.38	E
5	−0.02	5	0.14	−1.1	−1.09	0.66	1.42	EG
6	4.97	2	0.13	4.99	6.31	8.1	−0.5	G
7	1.04	−0	0.05	1.98	0.1	1.83	−1.01	G
8	−2.03	0	0.16	−0.04	0.03	0.11	2.78	DE, EK
9	−0.05	−0.88	−0.57	−0.18	1.26	5.27	−0.85	K
10	−0.04	−0	1.78	0.83	−0.69	−0.15	−0.73	K
11	3.69	1	3.01	1.09	3.84	0.28	8.09	K
12	−0.02	3	3.9	3.09	1.02	0.95	14.05	DK
13	0.1	−0	1.94	0.95	−0.96	−0.01	−1.02	KM
14	3.02	1	0.21	−0.53	−0.02	1.24	1.08	C
15	1.96	−0	0.22	0.01	0.05	−0.82	1.6	C
16	0.04	−0	−0.86	−1.07	−0.92	5.28	−0.47	E
17	0.45	−0	1	−0.01	0.58	−1.15	0.12	CI
18	0.97	1	−0.04	1.04	0.17	0.98	1.88	I
19	−0.07	1	0.1	−0.03	−0.07	0.54	0.54	C, CE, EI
20	2.45	−0	2.34	0.03	0.6	−0.25	−2.15	CE
21	0.19	−0	0.04	−0.02	0.37	−1.02	2.43	CF
22	2.12	−0	1.01	0.99	2.99	0.29	1.61	CJ, EJ
23	10.4	4.21	−3.91	1.14	0.7	7.6	11.86	E, CE, EJ
24	1.01	−1	0.13	−0.09	0.08	0.18	−0.29	EF
25	0.53	−0.14	2.57	−0.11	0.18	3.74	−0.77	E
26	1.01	−0	1.07	0	0.04	0.18	0.7	E
27	1.09	−0.13	0.03	−0.03	0.12	0.32	−0.4	E
28	−2.03	−0	−2.01	0.02	0.15	4.82	2.05	E
29	0.85	3	0.1	1.04	8.07	−1.45	2.39	E
30	0.02	3	4.92	1.04	2.06	0.22	2.74	E
31	−1	−1	1.11	−0.05	0.07	0.65	2.23	E
32	−0.11	−0	0.07	0.04	0.01	0.48	2.51	E
33	0.02	−0	0.2	−0.06	0.04	0.68	1.12	E
34	0.05	−0	0.04	0.01	0.01	0.13	4.75	E
35	16.6	13	5.85	−0.05	−0.29	10.99	35.9	E
36	−0.02	0	4.11	−0.03	3.07	3.1	7.76	E
37	4.01	−0	3.01	−0.08	1.1	1.02	0.93	JL, JN
38	2.03	−0	1.09	−0.04	−5.04	2.1	3.86	LN, NO
39	0.03	−0	0.04	0.07	0.06	0.34	4.46	L, O
40	5.6	0	−0.72	0.04	−0.68	0.91	−3.15	E
41	5.05	1	3.06	−0.05	−4.05	0.95	5.05	EJ, EL, EN, EO
42	4.01	−0	1	−0.01	−3.92	0.32	−0.4	CE
43	0.1	−0	1.37	−3.03	6.94	0.83	−1.2	C
44	−1	−0	3.97	−0.1	0.04	−1.09	2.17	E
45	−2.01	1	0.12	0.96	0.04	4.56	2.33	E
46	2.21	−0	1.53	0.09	−2.53	0.01	−0.3	CG
47	9.24	−0	−3.8	−0.04	−0.55	1.35	−4.21	E
48	−0.05	−0	1.27	−0.02	0.12	−1.8	1.48	E
49	12.99	3	−4.23	1.04	−8.46	2.51	1.15	E
50	0.08	10	14.99	8.05	6.08	5.79	4.0	BE
51	4.97	6	3.03	2.02	−0.17	0.91	0.24	E
52	8.89	0	−3.71	−0.07	−0.34	1.03	−3.81	E
53	7.69	3	5.51	3.02	3.03	8.41	3.34	E
54	8.93	−0	−3.66	−0.02	0.04	1.18	−4.48	EK, EM
55	3.89	−0	1.27	−0.44	−1.65	1.86	−0.93	EM
56	11.3	1	−0.98	1.91	2.56	2.5	−3.29	E
57	0.97	0	3.23	0.11	0.08	0.01	0.6	B
58	−0.01	5	0.16	0.01	0.18	9.49	2.18	A
59	0.1	1	−0.89	2.01	−1.11	0.16	2.74	AB

* Minus signs, including −0, indicate incongruity at that node for that locus.

**Figure 1:**
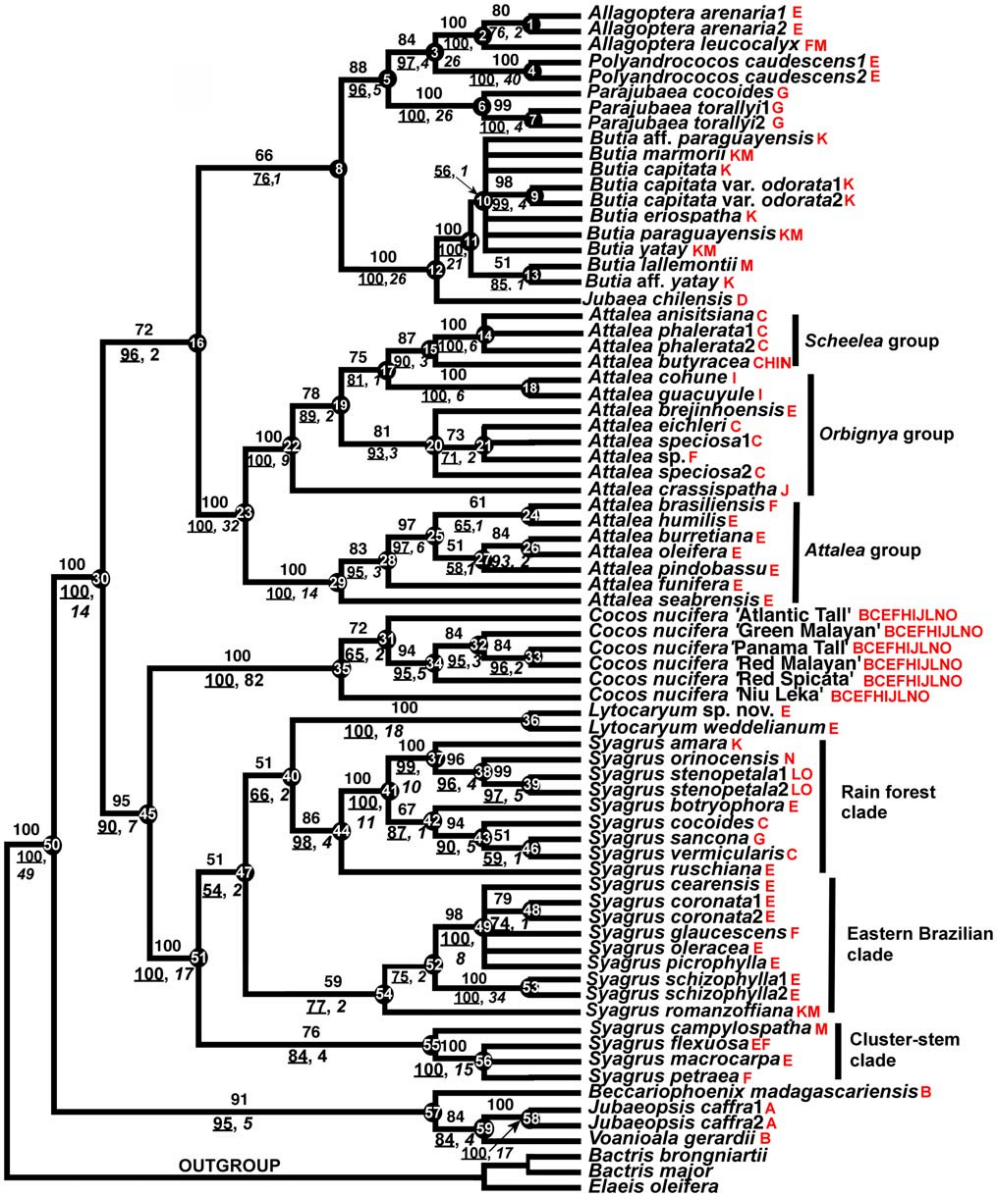
Strict consensus tree of equally parsimonious trees found by heuristic maximum parsimony analysis of seven combined WRKY loci sequences aligned across Areceaceae tribe Cocoseae subtribe Attaleinae. Numbers above branches are bootstrap support percentages. Numbers below branches are ML bootstrap support (underlined) and non-partitioned decay indices (italic). The numbers at each node refer to Table 2, which see for partitioned decay indices. Letter designations in red are area distributions of terminal taxa: A = South Africa, B = Madagascar, C = Amazonas (north, east, south), D = Chile, E = Eastern Brazil, F = Central Brazil, G = Andes, H = Central America, I = Mexico, J = West Indies, K = Southern Brazil, L = Northern South America, i.e., Caribbean coastal Venezuela and Colombia, French Guiana, Guyana, Surinam, M = Argentina-Paraguay-Uruguay, N = Colombia-Venezuela (*llanos* region), and O = western Amazonas.

† refer to Fig. 1 for node numbers.

**A = South Africa, B = Madagascar, C = Amazonas (north, east, south), D = Chile, E = Eastern Brazil, F = Central Brazil, G = Andes, H = Central America, I = Mexico, J = West Indies, K = Southern Brazil, L = Northern South America, i.e., Caribbean coastal Venezuela and Colombia, French Guiana, Guyana, Surinam, M = Argentina-Paraguay-Uruguay, N = Colombia-Venezuela (*llanos* region), and O = western Amazonas.

#### Super-matrix (concatenated) analysis

The combined sequence matrix consisted of 5831 total characters of which 974 were parsimony informative (17%). Heuristic search with parsimony found 446 equally most parsimonious trees of length = 2614, CI = 0.73 and RI = 0.87. Combining all seven WRKY loci yielded the most fully resolved trees and the highest bootstrap support (Fig. 1), both with MP and ML (the second, under-lined BP value between parentheses in the following discussion is that for ML). Partitioned Bremer (decay) indices (DI) (Table 2) indicates the relative contribution of each locus.

**Figure 2:**
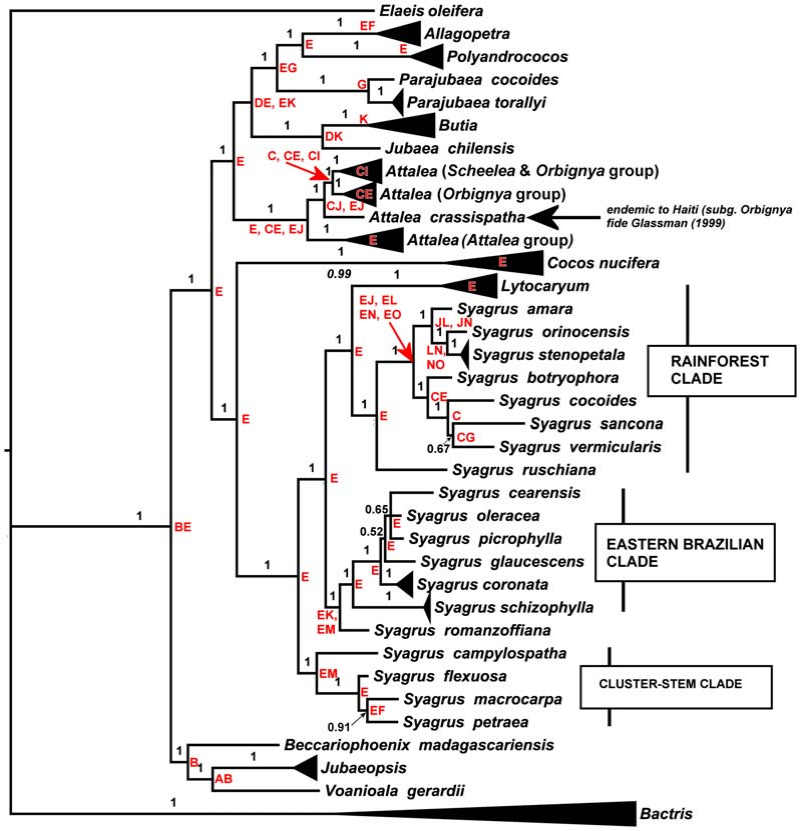
Majority rule consensus of 12,500 trees after burn-in sampled from mixed model (partitioned) Bayesian analysis of seven combined WRKY loci sequences aligned across Areceaceae tribe Cocoseae subtribe Attaleinae with MrBayes. Numbers above branches are posterior probability scores, i.e., the proportion of tree within which that clade was resolved. Letter in red at nodes are ancestral area optimizations as determined by dispersal-vicariance analysis: A = South Africa, B = Madagascar, C = Amazonas (north, east, south), D = Chile, E = Eastern Brazil, F = Central Brazil, G = Andes, H = Central America, I = Mexico, J = West Indies, K = Southern Brazil, L = Northern South America, i.e., Caribbean coastal Venezuela and Colombia, French Guiana, Guyana, Surinam, M = Argentina-Paraguay-Uruguay, N = Colombia-Venezuela (*llanos* region), and O = western Amazonas. Ambiguous area optimizations at a node are separated by commas.

The ML (Fig. S4) tree was essentially identical to the parsimony consensus (Fig. 1), although the terminals were more fully resolved. In general, BP was higher with ML (Table 3, Fig. S4), though generally with weak support wherever a polytomy appears in the parsimony strict consensus (Fig. 1). Bayesian analysis of the combined data matrix (Fig. 2) was also congruent with parsimony and ML, except for the lack of resolution for *Bactris* (Bactridinae). Most clades had posterior probability (PP) scores = 100%. The only clades with <90% were at several terminal nodes in *Syagrus*.

**Table 3:** Results of maximum likelihood analyses of subtribe Attaleinae with seven WRKY loci.

Locus	Nt substitution model	Nt frequencies	Nt substitution rates	Tree score	Genera* resolved as monophyletic (monotypic excluded) (BP†)	Clades Resolved (BP*)
WRKY02	TIM+G	T: 0.262, C: 0.204, A: 0.283, G: 0.251	TC: 0.358,TA: 0.129, TG: 0.091, CA: 0.091, CG: 0.129, AG: 0.201	−3179.53	ALL (100), ATT (88), BUT (98), LYT (52), PAR (98)SYA (99, if LYT included),	ALL-POL (54), BUT-JUB (59), ATT-(BUT-JUB) (84), (JUBA-VOA)-BEC (63), ((JUBA-VOA)-BEC)-COC (60), (((JUBA-VOA)-BEC)-COC)-PAR (60)
WRKY06	HKY+G	T: 0.358, C: 0.148, A: 0.298, G: 0.195	TC: 0.323, TA: 0.088, TG: 0.088, CA: 0.088, CG: 0.088, AG: 0.323	−2527.82	ALL (100), ATT (92), BUT (79), PAR (94), SYA (100, if LYT included))	ALL-PAR (87), (ALL-PAR)-POL (98), ATT-COC (64), ((ALL-PAR)-POL)- (ATT-COC)) (92), BUT-JUB (97), JUBA-VOA (77)
WRKY07	TrN+G	T: 0.286, C: 0.180, A: 0.297, G: 0.236	TC: 0.350, TA: 0.083, TG: 0.083, CA: 0.127, CG: 0.127, AG: 0.229	−3378.32	ALL (100), (BUT (86), LYT (83), PAR (100)	ALL-PAR (100), BUT-JUB (91), (BUT-JUB)-POL (94), COC-SYA “Amazonian” subclade (68), JUBA-BEC (69), (JUBA-BEC)-VOA (92)
WRKY12	TVM+G	T: 0.303, C: 0.177, A: 0.280, G: 0.240	TC: 0.323, TA: 0.033, TG: 0.087, CA: 0.116, CG: 0.119, AG: 0.323	−2136.60	ALL (86), ATT (90), BUT (79), PARA (96)SYA (87 if LYT included),	ALL-POL (97), BUT-JUB (93), (BUT-JUB)-PAR (69), (LYT-SYA)-ATT (66), ((LYT-SYA)-ATT)-COC (60), JUBA-VOA (96), (JUBA-VOA)-BEC (69)
WRKY16	HKY+G	T: 0.385, C: 0.168, A: 0.302, G: 0.145	TC: 0.308, TA: 0.096, TG: 0.096, CA: 0.096, CG: 0.096, AG: 0.308	−2666.74	ALL (100), BUT (100), LYT (99), PARA (99), SYA (69)	ALL-POL (67), ATT-COC (91, ATT paraphyletic with COC), JUB-PAR (63)
WRKY19	HKY+G	T: 0.340, C: 0.234, A: 0.267, G: 0.159	TC: 0.276, TA: 0.112, TG: 0.112, CA: 0.112, CG: 0.112, AG: 0.276	−2793.87	ATT (94), BUT (51), PAR (100),SYA (98, if LYT included)	ALL-POL (88), BUT-JUB (78), COC-SYA (including LYT; 90), (ALL-POL)-(BUT-JUB) (79), ((ALL-POL)-(BUT-JUB))-PAR (79)
WRKY21	HKY+G	T: 0.359, C: 0.162, A: 0.323, G: 0.155	TC: 0.271, TA: 0.114, TG: 0.114, CA: 0.114, CG: 0.114, AG: 0.271	−6021.78	ALL (98), ATT (100), BUT (100), LYT (100), PAR (100), SYA (90)	ALL-POL (98), (ALL-POL)-PAR (51), BUT-JUB (100), JUBA-VOA (93), ((ALL-POL)-PAR)-SYA (73), ((BUT-JUB)-(JUBA-VOA)) (62)

* ALL = *Allagoptera*, ATT = *Attalea*, BEC = *Beccariophoenix*, BUT = *Butia*, COC = *Cocos*, JUB = *Jubaea*, JUBA = *Jubaeopsis*, LYT = *Lytocaryum*, PAR = *Parajubaea*, POL = *Polyandrococos*, SYA = *Syagrus*, VOA = *Voanioala*.

† BP = bootstrap %.

The monophyly of all genera of the Attaleinae is supported by the combined WRKY data matrix (Fig. 1), with the exception of *Syagrus*, which is paraphyletic with *Lytocaryum*. The very well-supported subtribe (100/100% BP; support for the Attaleinae crown node was validated by running successive bootstrap analyses with just *Elaeis oleifera* and then the *Bactris* spp. as the designated outgroup) consists of three clades. The first represents the African genera (91/95% BP), within which *Becarriophoenix* is sister to a *Jubaeopsis*/*Voanioala* clade (84/84% BP). Partitioned Bremer support for this clade (Table 2) is positive (6 loci) or neutral (1 locus) for all seven loci, and universally positive for its sister relationship to the rest of the Attaleinae. The African clade is sister to the American clade, which resolves as two monophyletic groups. The better supported of the two (95/90% BP) consists of *Cocos* strongly supported as sister to *Syagrus* inclusive of *Lytocaryum* . Only WRKY2 is incongruent with this resolution (Table 2). Four of seven loci resolved *Lytocaryum* as nested within *Syagrus* (Figs. S1–4). The second, less well-supported (72/96% BP), clade of American genera unites *Butia* and *Jubaea* in a clade (100/100% BP) that is sister to a fairly well-supported clade (88/96% BP) of *Allagoptera* (100/100% BP), *Parajubaea* (100/100% BP) and *Polyandrococos*. *Allagopetra* and *Polyandrococos* form a clade with moderate to high support (84/97%). A monophyletic *Attalea* (100/100% BP) is sister to the rest of this clade. Within *Attalea*, monophyletic subg. *Attalea* (100/100% BP) and *Scheelea* (75/81% BP) subclades are resolved, while the *Orbignya* group appears paraphyletic to *Scheelea* (Fig. 1). Substructure in the *Syagrus* clade includes a “rain forest” group (86/98% BP), uniting species from Amazonas, the Andean foothills, the Caribbean and the Atlantic rain forest of Brazil (Fig. 1), to which *Lytocaryum* has a weaker sister relationship. An “Eastern Brazilian” clade is resolved as sister to the rain forest group. Finally, the outlying clade in the genus (76/84% BP) unites three clustering (multiple stems) species with the solitary-stemmed *S. macrocarpa*.

#### Reconciling gene and species trees

Reconciling individual gene trees to the species trees (generated from the combined analysis) necessitated significant costs in terms of deep coalescence events (Table 4). Locus WRKY21 had the lowest cost, followed by WRKY2 (these loci also were the most highly congruent based on the ILD). The greatest number of deep coalescence events, 82, occurred with WRKY12.

**Table 4:** Costs of reconciling each of seven WRKY loci gene trees (5 trees each) with a sample of equally parsimonious trees (5) found with the combined sequence matrix (“species” trees).

Locus	Deep Coalescence Events (mean of 5 gene trees against 5 species trees±SD)
WRKY2	57.8±1.6
WRKY6	74.0±4.7
WRKY7	77.6±6.5
WRKY12	81.5±8.6
WRKY16	72.6±4.6
WRKY19	68.3±6.3
WRKY21	49.0±2.8

Twenty-five heuristic searches in GeneTree [37], [38] found a total of 190 species trees. The strict consensus of these trees (Fig. S5, nodes collapsed to the generic level) resolves all genera as monophyletic, except for a paraphyletic *Syagrus* in which *Lytocaryum* is embedded (some of the gene trees used as input resolved *Lytocaryum* as sister to *Syagrus*). The American and African clades are resolved. In the African clade, *Jubaeopsis* and *Voanioala* are sisters, with *Beccariophoenix* subtending, as with MP, ML and BA (Figs. 1, 2). In the American clade, *Jubaea* is sister to *Butia*, *Polyandrococos* and *Allagoptera* also form a clade, but there is no further internal resolution. The GeneTree results must be approached with caution, as the five trees used for each locus represent only a small fraction of the total number of fully-resolved trees found for each.

#### Divergence time estimates

**Figure 3:**
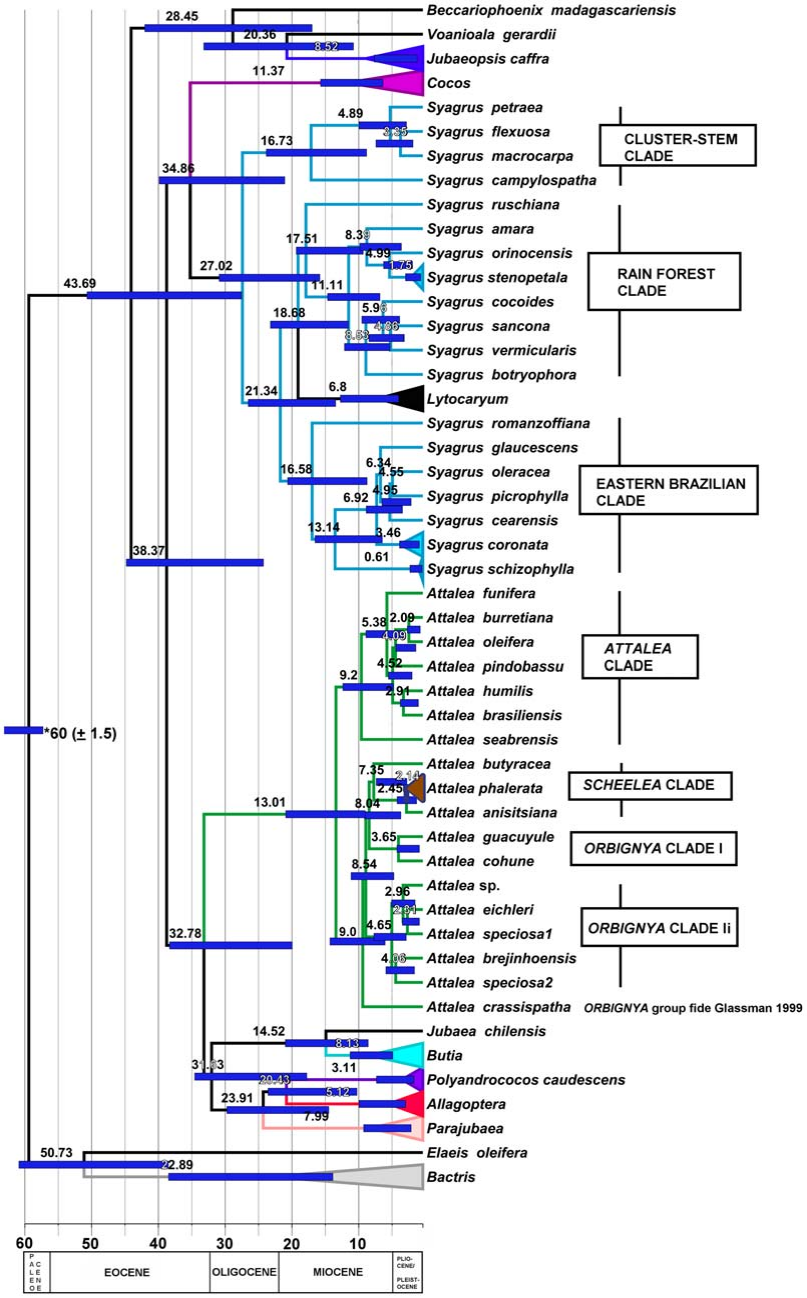
Maximum clade credibility chronogram generated from 21,000 trees samples by non-partitioned Bayesian analysis of seven combined WRKY loci sequences aligned across Areceaceae tribe Cocoseae subtribe Attaleinae with BEAST, drawn with branches proportional to absolute age in millions of years. Numbers are mean node ages; blue bars at nodes represent range of 95% HPD for the age estimate. Triangles indicate collapsed generic, sub-generic or species nodes. * = calibration point.

The maximum-clade-credibility tree (Fig. 3) produced by BEAST [39] was identical in the topology of the Attaleinae to the maximum likelihood tree (Fig. S4) and the majority rule consensus of the 12,500 trees sampled from the mixed model (partitioned) MrBayes analysis (Fig. 2), with the exception of a few taxa that terminate zero length branches, and some lower PP at some terminal clades. Table 5 provides the age estimates of the important nodes, and mean dates are mapped on a chronogram derived from the maximum-clade-credibility tree (Fig. 3). The most recent common ancestor (MCRA) of the African and American clades is estimated at 43.7 MYBP, with a 95% highest posterior density (HPD) range of 27–50 (Table 5). Crown age for the American Attaleinae is 38.4 MYBP (23.9–44.4 95% HPD), while that of the modern African genera is 28.6 MYPB (10.4–32.8 95% HPD). Crown clade ages for the two main clades of American Attaleinae are ca. 33 and 35 MYBP, respectively, the latter node being the MCRA of *Syagrus* and *Cocos nucifera*. *Syagrus* (including *Lytocaryum*) would appear to be the oldest genus in the American Attaleinae, with a crown age of 27 MYBP (15.4–30.5 95% HPD), while the equally speciose *Attalea* is relatively young at 13 MYBP (8.6–20.5 95% HPD). The three main clades of *Syagrus* s. s. share crown node ages of ca. 16.6–17.5 MYBP.

**Table 5:** Estimated divergence dates for selected clades within Cocoseae subtribe Attaleinae.

Clade	Mean (MYBP)	95% HPD* lower bound	95% HPD upper bound
Stem node Attaleinae (calibration)	60.0	57.0	62.8
TMRCA** Attaleinae	43.7	27.2	50.3
TMRCA African Attaleinae	28.5	16.6	41.6
TMRCA American Attaleinae	38.4	23.9	44.4
TMRCA *Allagoptera*/*Polyandrococos*	20.4	9.9	23.3
TMRCA *Attalea*	13.0	8.6	20.5
TMRCA *Butia*	8.1	4.5	10.9
TMRCA *Butia*/*Jubaea*	14.5	8,2	20.6
TMRCA *Cocos/Syagrus*/*Lytocaryum*	34.9	20.7	39.5
TMRCA *Syagrus* (inc. *Lytocaryum*)	27.0	15.4	30.5
TMRCA *Parajubea*/*Butia*/*Jubaea*/*Allagoptera*	31.6	17.4	34.2
TMRCA Outgroup	50.7	41.1	63.3

* HPD = Highest Posterior Density.

**TMRCA = time of most recent common ancestor.

#### Biogeographic analysis

Dispersal-vicariance optimization places Madagascar (B) and Eastern Brazil (E) as the ancestral distribution for the Attaleinae, with subsequent vicariance between the two hemispheres (Fig. 2), and one dispersal to South Africa (*Jubaeopsis*). Both major clades of the American Attaleinae remained restricted to eastern Brazil until an Eocene/early Oligocene range extension to coastal Chile or Southern Brazil by the MCRA of *Allagoptera, Butia*, *Jubaea, Polyandrococos* and *Parajubaea*, and Miocene dispersals to Amazonas and the West Indies (*Attalea*). Range extensions of *Syagrus*, first into contiguous Southern Brazil and Argentina-Paraguay, did not occur until the mid-Miocene (Fig. 2, 3), with even later dispersal of the genus into Amazonas, Northern South America, and the West Indies. Despite its subsequent pantropical distribution, the crown node of *Cocos nucifera* is unambiguously positioned in eastern Brazil.

#### Leaf anatomy

**Figure 4:**
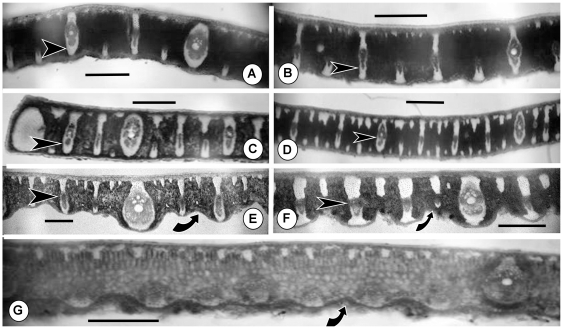
Freehand, unstained transverse sections of leaflets of *Allagoptera*, *Parajubaea* and *Polyandrococos*. A. *Allagopetra arenaria*. B. *A. brevicalyx*. C. *A. campestris*. D. *A. leucocalyx*. E. *Parajubaea cocoides*. F. *P. torallyi*. G. *Polyandrococos caudescens*. Black arrows with white outlines (A–F) point to “girder”-like vascular bundles in *Allagoptera* and *Parajubaea*. Curved arrows indicate depressions in abaxial surface (E–G) characteristic of *Parajubaea* and *Polyandrococos*. Scale bars = ca. 0.5 mm.

As the results of our molecular analysis were examined, it became clear that a survey of leaf anatomical characters within Attaleinae in progress by one of us (L. Noblick) lent support to one of the clades resolved by the WRKY genes. *Allagoptera*, *Parajubaea* and *Polyandrococos* all have have nonvascular bundles attached to their hypodermis layers (Fig. 4), but only *Parajubaea* and *Allagoptera* have very distinctive, “girder”-like narrow vascular bundles that span the distance between the upper and lower surface of the leaflets Fig. 4A–F). These are absent in *Polyandrococos* (Fig. 4G). However, both *Parajubaea* and *Polyandrococos* have an irregular, undulating abaxial surface often with hairy depressions, which is less visible in some species of *Allagoptera*. (Fig. 4A, B)

## Discussion

### WRKY loci

WRKY transcription factors are predominantly plant-specific proteins, broadly distributed across the genome [40]. A single WRKY locus is found in the ancient eukaryote *Giardia lamblia* Kofoid & Christiansen and the mycetozoan *Dictylostelium discoideum* Raper, but are absent from the genomes of fungi and animals [40]. WRKY genes, members of the WRKY-GCM1 superfamily [41], [42], contain a highly conserved DNA binding domain about 60 amino acids in length composed of the conserved WRKYGQK sequence followed by a C_2_H_2_- or C_2_HC-type zinc finger motif. Eulgem et al. [43] classified them into groups and subgroups based upon the number and type of WRKY domains, additional amino acid motifs, and phylogeny resolved from 58 loci isolated from *Arabidopsis* (DC.) Heynh. Group 1 WRKY genes were defined by the presence of two WRKY domains, each of the C_2_H_2_-type zinc finger motif, with only the C-terminal WRKY domain actively binding DNA. Group 2 WRKY genes contained only a single WRKY domain, and were classified into subgroups a-e based upon additional amino acid motifs found outside the WRKY domain. Group 3 WRKY genes were defined by the presence of the C_2_HC-type zinc finger motif in the DNA-binding WRKY domain. Further annotation of *Arabidopsis* WRKY genes [44] and phylogenies of *Oryza* L. and *Arabidopsis* WRKY gene families have resulted in several minor modifications to the original classification scheme [40], [45]–[46]. WRKY proteins are involved in several diverse pathways [44], [47]–[49] including regulation of starch, anthocyanin, and sesquiterpene anabolism; seed development [48], [49]; trichome development; embryogenesis; and plant responses to both abiotic and biotic stresses [44].

A feature common to WRKY genes is interruption of the coding region of the highly conserved, DNA-binding functional WRKY domain (the C-terminal WRKY domain in Group 1 WRKY genes and the single domains of Groups 2 and 3) with an intron. The size and sequence of the intron varies in each gene, but its position is highly conserved within each group/subgroup [40], [42], [43], [45]. Variability present in the intron distinguishes among diverse WRKY loci isolated from a single species and, in many cases, allows for the design of primers specific to each [20], [21]. The ancient origin and evolutionary expansion of the WRKY family was confirmed by discovery of a single WRKY gene each in *D. discoideum*, *G. lamblia*, and *Chlamydamonas reinhardtii* Dangeard, several from the moss *Physcomitrella patens* (Hedw.) B.S.G. and the fern *Ceratopteris richardii* Brongn. [48] to over 100 in *Oryza sativa* L. [47]. This expansion was due primarily to large-scale duplications of entire genomic regions as a result of separate polyploid events [40], [48]–[52] throughout plant evolutionary time, rather than via tandem repeats, with a great deal of microsynteny retained even between evolutionarily distant plant species [53], [54]. Rapid diversification of WRKY genes predates the divergence of monocots and dicots [45], [55].

### WRKY loci and orthology/paralogy

Paralogy is the leading concern when using nuclear genes, especially members of multigene families. Paralogous sequences from gene duplication events due to unequal crossing over, replicative transpostition or ancient polyploidization events, when unrecognized, may lead to erroneous phylogenetic inferences [56], [57]. Several lines of evidence, both direct and indirect, argue against paralogy as an issue with WRKY loci, although it cannot be ruled out conclusively without linkage mapping of the loci. In *Oryza sativa*, of the 102 WRKY loci described from subsp. *indica*, 99 are unigenes; of the 98 copies characterized in subsp. *japonica*, 97 are unique [58]. For the seven loci chosen for this study, there was little indication, either by direct sequencing multiple individuals from a single species or from cloning, of paralogous copies for any of the individual WRKY loci within a single species, although allelic variation was detected (see Materials and Methods). Certainly, WRKY loci occurring in different groups, either in the Euglem et al. [43] classification or the more recent modification of Zhang and Wang [40] represent orthologs. Two of our loci belong to Group 2c (WRKY16 and WRKY19). These two loci were placed in different clusters of group 2c and each had greater identity with orthologs from *Theobroma*, *Persea* and *Oryza* than with each other. One locus (WRKY21) belongs to group 2b. Four of the loci belong to Group 1 (two WRKY domains). Two of these (WRKY6 and WRKY7) resolve in two distant clusters based on either the C- or N-terminus domains. Two loci (WRKY2, WRKY12), tended to cluster close together based on their conserved domains, thus raising the possibility that they could represent recent paralogs. Nonetheless, primers designed for each of these three amplifies a single product in the vast majority of species that we sampled, suggesting that their divergence occurred before the diversification of the Cocoseae. Further evidence of the independence of these loci is the fact that the sequences of all seven amplified from a single species could not be aligned except over portions of their highly conserved WRKY domains.

### Phylogeny of the Attaleinae

Gunn's [5] analysis with the nuclear gene *prk* from across all 20 genera of Cocoseae supported recognition of a “spiny” clade (monophyletic Bactridinae and *Elaeis*; the monotypic genus Amazonian *Barcella* Drude resolved as sister to the rest of the tribe), and a “non-spiny” clade, i.e., the Attaleinae. Asmussen et al.'s [10] four plastid gene analysis of the entire family also resolved these two clades of Cocoseae with 91% BP (spiny clade) and 71% BP (non-spiny) , but with less than 50% BP for the tribe as a whole. The monophyly of subtribe Attaleinae is unquestionable (Figs. 1–2). WRKY loci resolve the African genera as a distinct clade sister to the American genera (Figs. 1–2), and *Voanioala* and *Jubaeopsis* as sister genera. Gunn's [5] analyses either resolved this clade as paraphyletic (BA and ML), or as a polytomy with the American clade (parsimony). These three genera all depart from the *n* = 16 chromosome number that is characteristic of the American clade [59]. *Beccariophoenix madagascariensis* has *n* = 18, while *Jubaeopsis* (*n* = 80–100) and *Voanioala* (*n* = 298) have been suggested as autopolyploids [60]. The WRKY resolution (Figs. 1–2) suggests that Madagascar rather than mainland Africa was the point of origin of the clade, with subsequent vicariance to South Africa. This is further supported by the fact that both *Jubaeopsis* and *Voanioala* are polyploids, while *Beccariophoenix* has a chromosome number much closer to the American Attaleinae.

The “*Cocos* alliance” resolved by WRKY genes consist of only *Cocos nucifera*, *Syagrus* and *Lytocaryum* (embedded in *Syagrus*), positioned as sister to the “Attalea alliance” of all other American genera. As in Gunn's [5] study, the sister relationship of *Jubaea* and *Butia* is very well supported, congruent with their similar leaf [61] and root [62], [63] anatomy, but the novel clade positioning of *Attalea* as sister to *Butia*/*Jubaea* has high support only with ML and BA (Figs. 1–2).

The close relationship of *Lytocaryum* and *Syagrus*, inferred by previous studies with plastid [13] and nuclear [5] sequences, and evidenced morphologically as well [14], [16] is here further corroborated. As in Gunn [5], one of our loci (WRKY7) resolves a sister relationship specifically with *S. romanzoffiana* (Table 1; Fig. S1). *Syagrus ruschiana*, which is sister to all of the other “rain forest” clade species, is, like *Lytocaryum*, a plant of the Brazilian Serra do Mar, and also bears fruits that split at the tip with thin exocarp and mesocarp, much like *Lytocaryum*. The sum of the evidence to date suggests that *Lytocaryum* should be transferred to *Syagrus*, but the low bootstrap support for its exact position relative to *Syagrus* in our combined tree (Fig. 1) might argue for awaiting further data.

The robust monophyly of *Attalea*, formerly split into as many as six genera largely on the basis of male floral characteristics [64]–[66], is indisputably supported by the WRKY sequences (Figs. 1–2) – only two of the seven gene trees (WRKY7, WRKY16) fail to resolve the monophyly of this genus. The only Caribbean species in the genus, *A. crassispatha*, endemic to Haiti, is robustly sister to both a distinct *Scheelea* and two *Orbignya* subclades (Fig. 2). Glassman [66] placed this species in *Orbignya*. Within the monophyletic *Scheelea* clade, *A. anisitsiana* resolves consistently with two collections of *A. phalerata*, to which some workers have assigned it to synonomy [18], [65]. Similarly, *A. cohune* and *A. guacuyule*, the latter considered synonomous with the former in some accounts [18], [65], resolve robustly as sister species.

### The origins of the coconut

With three plastid genes [13], *Cocos* resolved as sister to *Attalea* H. B. K. in a weakly supported clade. In Gunn's study with the low copy nuclear gene *prk* [5], *Cocos* was positioned as sister to *Parajubaea* Burret, but only with ML. With MP or BA, its exact relationships were unresolved. Even a total evidence supermatrix/supertree approach, which included all molecular markers commonly used in palm systematics (13 in total), plus both morphological and RFLP datasets, did not help resolve the exact position of *Cocos* within the Attaleinae [19], placing the genus as sister to *Parajubaea* in a clade with only 52% BP. The WRKY consensus positions *C. nucifera* as sister to the South American genus *Syagrus*, and the clade has very strong support (Figs. 1–2), last sharing a common ancestor ca. 35 MYBP (Fig. 2, Table 5), though the crown node age of modern *Cocos* is estimated at only ca. 11 MYPB. The taxonomic history of *Cocos* and *Syagrus* has long been intertwined [5], [16], [67]. In the first edition of Genera Palmarum [16], this relationship was explicitly noted, but was expunged from the second edition [14], based on Gunn's [5] weak resolution of *Cocos* as sister to *Parajubaea* with ML. Our analysis presents the strongest evidence to date for a close phylogenetic relationship between *Cocos* and *Syagrus*, and that the biogeographic ancestry of the coconut, regardless of its subsequent etnhobotanical history, is firmly rooted in South America. Although only two loci on their own explicitly support the sister relationship to *Syagrus* (WRKY7: Figs. S1, S3; WRKY19: Figs. S2, S4), only a single locus (WRKY2) in the combined analysis has incongruent partitioned Bremer support at the crown node for *Cocos*–*Syagrus*/*Lytocaryum* (node 45 in Fig. 1 and Table 2). The crown node of the six coconut genotypes included in our analysis terminates a branch 110 steps in length, one of the longest on the trees, raising the specter of long branch attraction (LBA) [68]. Likelihood, which is less sensitive to LBA [68] also robustly supports this resolution, as do both our partitioned and non-partitioned Bayesian analyses. We tried several parsimony approaches that have been suggested to test for the presence of LBA [68]: i.e., outgroup exclusion, and removing all or some *Syagrus* and/or *Lytocaryum* sequences from the alignment. *Cocos* still resolved as sister to *Syagrus* (inc. *Lytocaryum*) when outgroups were removed. When *Syagrus* and *Lytocaryum* were removed from the alignment, *Cocos* resolved as the first branch after the African clade and thus sister to the remaining American Attaleinae. With *Syagrus* excluded, *Cocos* resolved as sister to *Lytocaryum*. With even just a single species of *Syagrus* included in the analysis (we successively included just one species from each of the three main *Syagrus* subclades), *Cocos* resolved as its sister. We believe that the relationship between *Cocos* and *Syagrus* resolved by seven concatenated WRKY gene alignments is real, considering our substantial depth of sampling of the Attaleinae, and that the number of steps as well as the appreciable time duration (>20 MY) between the stem and crown nodes of *Cocos* reflects intervening extinction events.

This resolution might indicate that an Atlantic dispersal of the progenitors of the coconut is likely, perhaps along now submerged mid- to south Atlantic stepping stones, since the diversity of *Syagrus* is concentrated in Eastern Brazil (Figs. 1–2). But as this event precedes the Andean orogeny (Fig. 2), a Pacific coast dispersal from a broadly distributed lowland rain forest ancestor of *Syagrus* and *Cocos* cannot be ruled out. The resolution of the South Pacific coconut variety ‘Niu Leka’ as sister to all other cultivars would support the latter scenario as well, and is congruent with a Pacific Ocean dispersal scenario for coconut [2].

Unfortunately, beyond the sister relationship of ‘Niu Leka’ to all other varieties, the well-resolved clade of coconut cultivars, while demonstrating the utility of WRKY loci at infra-specific levels, can not be interpreted strictly because SSR studies indicate that three of the six individuals (‘Atlantic Tall’, ‘Pacific Tall’, and ‘Red Spicata’) used in this paper were introgressed with other cultivars [69]. However, the degree of resolution suggests that WRKY loci could be successfully applied to a phylogeographic study of *Cocos nucifera*.

### The *Allagopetra*-*Parajubaea*-*Polyandrococos* clade

The most surprising clade is that which unites *Parajubaea* with *Allagoptera* and *Polyandrococos* . Gunn's [5] analysis of *prk* sequences embedded *Polyandrococos* within *Allagoptera*, and Dransfield et al. [17] formally transferred the monotypic genus into *Allagoptera*. *Polyandrococos* is sister to *Allagoptera*, rather than embedded within it as Gunn [5] resolved with *prk*, thereby rendering Dransfield et al.'s [17] transfer of *Polyandrococos* to *Allagoptera* equivocal. Both *Allagoptera* and *Polyandrococos* have spicate inflorescences and share similar pinnae phylotaxy [16]. The Andean *Parajubaea*, still uncertainly consisting of 1–3 species [70], [71] has been variously treated as synonymous with *Jubaea* [72], *Allagoptera* [73] and *Polyandrococos* [74]. *Parajubaea* was aligned with *Butia*, *Jubaea* and *Syagrus* by Uhl and Dranfield [16]. Leaf anatomy (Fig. 4) supports the relationship among *Allagoptera*, *Parajubaea* and *Polyandrococos*.

### Biogeographic and paleohistorical implications

Plant species exchange between Africa and South America continued after direct connections were severed in the late Cretaceous, at least through the early Paleocene, possibly via the Walvis Ridge/Rio-Grande Rise and Sierra Leone Ridges [75]–[77]. It was during the Paleocene that palms appear to have diversified greatly in Northern South America [78], [79], and the late Paleocene is where the stem age of the subtribe falls. In the early Eocene, fossil palm pollen was reduced in abundance in South America, perhaps due to climatic changes that occurred at the Paleocene/Eocene boundary but with little apparent loss of diversity [79]. A date of ca. 43.7 MYBP (Table 5, Fig. 3) for the MRCA of the African and American Attaleinae is similar to Gunn's (2004), estimate with three of her four fossil calibration points (∼43 MYPB; any further comparison of our age estimates to Gunn (2004) is difficult due to the incongruent resolution of generic relationships between our trees and hers). This predates the terminal Eocene global cooling event, which for reasons unknown had little effect on the South American flora, including palms, but resulted in massive extinctions in west Africa, very notably of palms [79]. However, the broad 95% HPD range (27.2–50.3 MYBP) at this node makes paleohistorical interpretation difficult. Thus, modern African representation of the Attaleinae is restricted to three relicts: a monotypic genus on the mainland (*Jubaeopsis*), and two Madagascar endemic genera (*Beccariophoenix*, 2 spp., and *Voanioala*, monotypic) that are both high polyploids, *Voanioala* with the highest chromosome number known in the monocotyledons [60].

Establishment of the two major clades of American Attaleinae on the other hand does appear congruent with the terminal Eocene cooling event (38.4 MYBP Table 5, Fig. 3), with subsequent cladogenesis in the Oligocene. Area optimization (Fig. 2) indicates that the ancestral American Attaleinae were restricted to Eastern Brazil during this time period. The MCRA of *Allagoptera* (including *Polyandrococos*), *Butia, Jubaea* and *Parajubaea* had dispersed to southern Brazil, the Andes and coastal Chile by the early Oligocene (Figs. 2, 3). The latter two may have been long distance dispersal events, as *Jubaea* consists of a single species of limited range in the central Chilean coast range, while *Parajubaea* is known only from cultivation in Andean Ecuador and Colombia (*P. cocoides*) and in the wild from the Andes of southern Bolivia (*P sunkha* and *P. torallyi*) [70], [71]. Alternatively, these rare taxa may represent relicts of a once more broadly distributed lineage that suffered massive extinction. A vicariant event splitting the the MRCA of *Jubaea*/*Butia* and *Parajubaea*/*Allagoptera* (including *Polyandrococos*) in the proto-Andean region at this time is congruent with a Pacific marine incursion known as the Western Andean Portal (WAP) or Guayaquil gap, which a number of studies have proposed took place from the Eocene to the mid Miocene [80]–[85], effectively disrupting exchange between the northern and southern Andean regions. The late Oligocene-early Miocene divergence between the Andean *Parajubaea* and the eastern Brazilian *Allagoptera* (including *Polyandrococos*) corresponds to the flooding of western Amazonia caused by the uplift of the Eastern Cordillera of the Central Andes in the early Miocene and subsequent Caribbean marine incursion to the north [86]–[90]. An enormous system of long-lived lakes and wetlands (“Lake Pebas” or the “Pebas Sea”) was formed that provided a significant barrier to dispersal from eastern South America to the Andes [86]–[90] that lasted until the Late Miocene. The divergence of *Jubaea* and *Butia*, however, appears to have occurred considerably later (14.5 MYBP), perhaps as the central Andes rose to sufficient elevation to obstruct dispersal or fragment a broad ancestral range of their MRCA.

Based on the estimated dates, much of the subsequent diversification in the Attaleinae can be attributed to the Andean uplift from late Miocene through the Pliocene [91]–[93], and Pleistocene fluctuations in the extent and location of rain forest and seasonally dry climates in South America [94], [95]. Species level divergences in *Attalea*, *Butia* and *Syagrus* are concentrated within the last 10 MYBP, much as Richardson et al. [96] determined for *Inga* Mill. (Fabaceae).

Other than the pantropical *Cocos nucifera*, the only two species of Attaleinae found in the Caribbean are both endemics: *Attalea crassispatha* (9 MYBP) in Haiti and *Syagrus amara* (8.4 MYBP) in the Lesser Antilles with essentially contemporaneous late Miocene divergence dates from their respective congenors.

WRKY genes in combination present the most fully resolved and well-supported phylogeny of the palm subtribe Attaleinae, and indicate a likely sister relationship between *Cocos* and *Syagrus*. Branch age estimates of our phylogeny are, in many cases, congruent with known paleohistorical events in South America. The biogeographic and morphological congruence that we see in the resolution of the larger genera such as *Attalea* and *Syagrus* (Figs. 1–2) suggest that WRKY loci are informative markers for investigating the phylogenetic relationships of Cocoseae, and should be tested further in the tribe, and perhaps other tribes of Arecoideae as well.

## Materials and Methods

### Sampling

DNA was isolated from living accessions of 75 taxa (Table S1), mostly in cultivation at the Montgomery Botanical Center and the USDA-ARS-National Germplasm Repository: 72 from all genera of subtribe Attaleinae, and, as outgroups, two species of *Bactris* (subtribe Bactridinae) and *Elaeis oleifera* (Elaeidinae). The strategy for larger genera was to sample evenly from all recognized geographic or morphological groups. Multiple individuals were sampled for several species in several genera as a consistency check.

### DNA extraction

DNA was extracted from silica gel dried leaf samples using the BIO101 kit as described in Mauro-Herrera et al. [27]. The quantity of DNA isolated was assessed with a GeneQuant pro RNA/DNA calculator (Amersham Pharmacia Biotech, Piscataway, N.J.). Isolated DNA was stored at −80°C until use.

### WRKY gene isolation

By using a degenerate primer pair [20], [21], 21 WRKY sequences were isolated from *Cocos nucifera* [22]. Of these, 12 were of sufficient size (>400 bp) to potentially yield a significant number of phylogenetically informative base substitutions, and specific primers were designed to maximize read length [the original primers of Mauro-Herrera et al. [22] were designed for short fragments flanking single nucleotide polymorphisms (SNPs)]. Ultimately, seven loci (Table S2) were selected that amplified a single product and which were able to be directly sequenced. The original sequences from *C. nucifera* were subjected to BLAST analyses against the non-redundant database at GenBank [97] and conceptual amino acid sequences of our eight loci were aligned with *Arabidopsis thaliana*, *Theobroma cacao*, *Oryza sativa*, and *Persea americana* WRKY proteins using ClustalX [98] to determine to which WRKY subgroup they belonged and to eliminate likely paralogs. Loci were chosen that had greater identity with orthologs from unrelated species than with orthologs from *Cocos nucifera*.

### DNA Amplification and sequencing

Amplifications contained: 0.200 nM forward and reverse primer, 200 µM dNTPs, 1 mg/ml bovine serum albumin, 1 x amplification buffer with 2 mM MgSO_4_, 0.025 U/µl reaction volume Taq DNA Polymerase (New England Biolabs, Ipswich, MA) and 10 ng of template DNA brought to a total volume of 15 µls with nuclease-free H_2_0. Amplifications were conducted using PTC-225 thermalcyclers (MJ Research, Waltham, MA). Conditions were: 95°C, 2 min; [95°C, 30 s; 57–64°C, 60 s; 72°C, 60 s] x 35 cycles; 72°C, 10 min; 4 C, hold. Amplification success was determined by agarose gel electrophoresis in 1.2% agarose, 0.5 x TBE buffer, ethidium bromide-stained, and visualized with UV light. Amplifications were treated with Exonuclease I and Shrimp Alkaline Phophatase to remove any unincorporated PCR primers and dNTP's, ethanol precipitated, and resuspended in sterile H_2_O. Direct sequencing was done in both directions on 1–2 µl of the treated amplification product with either the forward or reverse primer used for the initial amplification. All sequencing was done by capillary electrophoresis on an ABI 3100 or 3730 Genetic Analyzer using the BigDye Terminator Cycle Sequencing Ready Reaction Kit v3.1 (Applied Biosystems, Foster City, CA).

In the preliminary screening, seven WRKY loci produced a single band migrating at the expected mobility upon gel electrophoresis for each of three taxa amplified. Direct sequencing of the amplification products of most samples (Table S1) gave clean, clear signals with little or no noise for 60–100% of the taxa sampled. In some cases, double peaks gave evidence of allelic variation, and these were coded as ambiguities.

All sequences have been deposited in GenBank (Table S1) and assigned the following accession number ranges: WRKY2: FJ956927 - FJ956996, WRKY6: FJ957069 - FJ957142, WRKY7: FJ957143 - FJ957215, WRKY12: FJ957216 - FJ957283, WRKY16: FJ957284 - FJ957353, WRKY19: FJ957354 - FJ957428, WRKY21: FJ956997 - FJ957068.

### Cloning

Cloning was necessary for several taxa for six of the seven loci (Table S1). For pre-cloning PCR, AmpliTaq (Applied Biosystems, Foster City, CA) was used instead of NEB Taq polymerase. PCR products were cloned into pGEM-T vector, and the vector was transformed into JH109 High Efficiency Competent Cells following the instructions of the manufacturer (Promega, Madison, WI). Colonies were transferred to 96-well plates and incubated overnight at 37°C in SOC media with 100 µg/ml ampicillin. Transformed cells were lysed by resuspending the pelleted cells in 50 µls of 10 mM Tris-HCl pH 8.0. One µl of this was used as templates for PCR to confirm insert size on an agarose gel, and the PCR product was also used for the cycle sequencing reaction.

In most of the cases where cloning was necessary, clones showed allelic variation at SNPs or microsatellite repeats within introns, but consistently resolved as sisters with 100% BP when incorporated into the aligned matrix. For these, which constituted the majority, we used consensus sequences in our final alignments. Several species of *Attalea*, *Butia* and particularly *Syagrus* exhibited small indel polymorphisms among the clones, often evidence of interspecific hybridization, which has been reported in these three genera [99]–[101]. We noted that low frequency clones resolved at times with other species in their respective genera. For the present study, these clones were dropped. We plan to investigate this issue further with broader sampling.

### Alignment

Sequences were aligned using MAFFT [102], [103] with subsequent manual editing in Sequencher™ 4.8 (Gene Codes Corporation, Ann Arbor, MI). The aligned lengths (Table 1) ranged from 658 nt (WRKY16) to 1277 nt (WRKY21).

### Phylogenetic analyses

Aligned sequences for the seven WRKY loci were analyzed separately and in combination using MP with PAUP* v. 4.0b10 [104], and with two model-based approaches, ML, utilizing TreeFinder [105] and, for the combined analysis only, BA, with MrBayes v. 3.1.2 [106], [107]. Best fit nucleotide substitution model was determined for each gene region with KAKUSAN v.3 [108], which also generates input files for these two programs. Best fit models were evaluated using the corrected Akaike Information Criterion (AICc) [109], [110] for ML and, for the BA, the Bayesian Information Criterion (BIC) [111]. Significance of model fit statistics was determined by Chi-square analysis. For ML and BA, a mixed model, retaining each partition's best fit nucleotide substitution model, was applied.

#### Parsimony

MP tree searches were heuristic, conducted under the Fitch (equal) weights [112] criterion with 1000 rounds of random addition, saving no more than 10 minimum length trees per search for swapping using tree branch reconnection. Tree branches were collapsed if the minimum length = 0. Gaps were coded as missing characters in the initial analyses, but were also coded with the program SeqState [113], using the simple coding (SIC) of Simmons and Ochoterena [114]. Before combining the data sets, we performed an incongruence length difference test (ILD = partition homogeneity test in PAUP* [26], [27]) on all pairwise combinations of loci to assess the degree of congruence between them. One hundred heuristic searches were conducted, each with 10 random addition replications, saving no more than 10 trees from each for TBR branch swapping. Internal support was determined by bootstrapping [115] (1000 heuristic replicates with simple addition, TBR branch-swapping, saving 10 trees per replicate). The cut-off BP value was 50%. A BP value >85% was considered good support, 75–85% was designated moderate support, and ≤ less than 75% as weak. For the combined analysis, both partitioned and non-partitioned Bremer (decay) indices [116] using TreeRot v. 3.0 [117] were also calculated (Table 2). One hundred heuristic searches with random addition sequence were implemented for each constraint statement postulated by TreeRot, saving no more than 10 trees per search. A minimum DI = 2 was considered to represent good support for a clade [118], [119].

#### Maximum likelihood

Treefinder [105] scripts generated by KAKUSAN [108] were used in part to conduct the maximum likelihood analysis. The first single search, using the best fit proportional model generated by KAKUSAN [108], was used to generate optimum substitution model and substitution rates in Treefinder [105], as well as a single tree. The optimum model and rates were specified for a second, unrestricted search in Treefinder [105], with the single tree produced from the first as a starting tree. One thousand replicates were run, with a search depth of two. ML bootstrap support was generated with 500 replicates, applying the same model and rates.

#### Bayesian analysis

Two parallel runs were performed in MrBayes, each consisting of four chains, one “cold” and three incrementally heated. *Elaeis oleifera* was designated as outgroup. Two and one half million Markov chain Monte Carlo (MCMC) generations were run, with convergence diagnostics calculated every 1000th generation for monitoring the stabilization of log likelihood scores. Convergence was also evaluated for each of the two parallel runs using Tracer v.1.4 [120], with half (1.25 million) of the generations as burn-in. Effective sample sizes (ESS) of all parameters were >100. Trees in each chain were sampled every 100th generation. A 50% majority rule consensus tree was generated from the combined sampled trees of both runs after discarding the first 50% (12,500 trees, 6250 each run).

### Gene tree incongruence testing

Different loci used to infer phylogenetic relationships of the same taxa often result in incongruent topologies [121], [122], due to such factors as horizontal gene transfer, gene duplication or loss, or deep coalescence [123], the latter leading to incomplete lineage sorting [123], [124]. As we believe that our seven loci are orthologous , deep coalescence events are the likely reasons for “hard” incongruence among our gene trees. We attempted to resolve a species tree from the seven gene trees with a method more powerful than concatenation of the matrices. We first tried the program BEST v. 2.2 [125], which uses a Bayesian hierarchical model to estimate the phylogeny of a group of species using multiple estimated gene tree distributions [126]. When, after 25 million generations (six weeks on a Pentium Xeon with 2 GB RAM), log likelihood scores had not stabilized, we concluded that our computing resources were insufficient for efficient use of the BEST program. We then turned to GeneTree v. 1.3.0 [37], which estimates a reconciled species tree with the lowest “cost” from multiple individual gene tree topologies [38], costs being defined as inferred gene duplications, losses, or deep coalescence events, depending on the gene family history. We generated new, fully dichotomized trees by running 500 random addition heuristic search replicates in PAUP* as described above, saving the 10 shortest trees from each for TBR swapping to a final limit of 1000. We then determined pair-wise tree-to-tree symmetric distances in PAUP* for each gene (as well as the trees from the combined analysis), then ran principle coordinate analysis (PCA) on the normalized pair-wise distance matrices using Multivariate Statistical Package (MVSP v. 3.13, Kovach Computing Services, Anglesey, Wales). Five trees that appeared broadly distributed in tree space were selected for each locus and the combined analysis as gene trees and species trees, respectively. We tested the fit of the gene trees to the species trees, and also generated *de novo* species trees by conducting 25 heuristic searches in GeneTree with random tree starting points, no constraints, and gene tree bootstrapping (the latter is the only way to get GeneTree to sample all of the trees provided to develop a species tree). A strict consensus tree was generated in PAUP* from the species trees found by GeneTree.

### Divergence time estimation

Molecular dating was carried out using BEAST 1.4.8 [44]. The method implemented in BEAST [44], [127] simultaneously estimates divergence times, tree topology and rates, thereby providing a clear advantage over previous relaxed clock methods [128] that estimate tree topology and divergence dates separately [129]–[131]. For this study we relied on a fossil fruit from northern Colombia assigned to the *Attaleineae* and dated to ∼60 MYBP [132]. Though the authors named this fossil as “cf. *Cocos*” [132], we believe that to assume homology of the impression to modern *Cocos nucifera* would be rash (the presence of germination pores in the endocarp–the most diagnostic fruit character for all of tribe Cocoseae [14], was not able to be confirmed in this fossil impression). It was thus conservatively placed at the stem node of the Attaleinae.

The ML tree, found with TreeFinder and rendered ultrametric using the program r8s [133], was used as starting tree for the BEAST runs. The likelihood ratio test (LRT) implemented in the program HyPhy [134] was used to assess whether a molecular clock could be applied to any of the loci, setting the optimal substitution model, rates and base frequencies determined by KAKUSAN [108]. Based on the results of the LRTs, a global molecular clock was rejected for all seven WRKY loci, however HyPhy detected evidence of local clock-rate rates in portions of each tree. As the dataset deviated from a strict molecular clock model, a lognormal non-correlated relaxed clock model was specified in BEAST, and a general time- reversible substitution model with gamma-distributed rate heterogeneity (GTR+G) was invoked.

In order to accommodate for calibration uncertainty, we applied a normal distribution as a prior to the calibration node within the BEAST analysis with a mean of 60 myr and standard deviation of 1.5 (effectively enclosing dates from 558-62 MYBP). Although normal prior distributions are used when dating trees under an indirect approach [135], we prefer this type of distribution because it does not place a strict minimum age on the calibration. Indeed, the actual dating of the fossil is also subject to uncertainty and by allowing the ages to vary around the mean of the distribution appears as a more realistic choice.

A total of 10 different runs of 10 million generations each were undertaken on the online cluster of the Computational Biology Service Unit from Cornell University (http://cbsuapps.tc.cornell.edu/beast.aspx). This cluster imposes a time limit of three days (72 hours) per analysis but allows several runs of the same analysis simultaneously. Analyses were undertaken by sampling every 1000th generation. Tracer v.1.4 [120] was used to check for convergence of the model likelihood and parameters between each run until reaching stationarity. The resulting log and tree files were then combined using LogCombiner. Results were considered reliable once the ESS of all parameters was above 100 (see results for the total number of generations). Branches with posterior probabilities (PP) below 0.8 were considered as weak, between 0.8 and 0.95 as moderate, and above 0.95 as strong.

All 10 runs in BEAST reached stationarity within the first 10,000 generations (because the ML starting tree was already near optimum), and all parameter estimates were consistent between runs. Runs were thus combined, after removing a burnin of 100 trees each (10,000 generations), into a single run of ca. 100 million generations. All parameters, including age estimates, reached acceptable ESS values and were thus deemed reliable (ESS>100). The tree files were combined after a burnin of 100 trees for each run. As the resulting combined tree file was too large to analyze in TreeAnnotator, combining of the different runs was redone. It was thus re-sampled at a lower frequency of every 5,000^th^ tree resulting in a file containing 20,000 trees sampled from the posterior and used to generate a maximum clade credibility phylogram in TreeAnnotator (Fig. 3).

The results presented in this paper are based on a non-partitioned analysis. A partitioned analysis (using the closest approximations in BEAST of the models applied in ML and BA, Table 3) never reached stationarity. However, in the past we have observed that partitioned and non partitioned data returned identical age estimations (Couvreur, unpubl. data).

### Biogeographic analysis

The biogeographic patterns inferred from our gene trees were assessed using the dispersal-vicariance method of analysis [136] as modeled by the program DIVA version 1.2 [137]. The program uses vicariance (i.e., allopatric speciation) in its optimization of ancestral distributions but takes into consideration dispersal and extinction events and indicates their direction [136], [137]. The most parsimonious reconstructions minimize such events. Unlike other biogeographic inference methods based on a strict vicariance model [138]–[140], DIVA does not restrict widespread distributions to terminals or limit ancestral distributions to single areas [137]. By allowing for dispersal and extinction as well as vicariance events within its model, DIVA does not impose adherence of area scenarios to a rigid “area cladogram.” It is thus much more amenable for biogeographic analysis within regions that have a complex paleogeological history, which a strict vicariance model cannot adequately address. Ancestral area optimizations in DIVA become less certain as the root node of the tree is approached. A weakness of the program is its assignment of nearly every area occupied by the terminal taxa in the tree to the more basal nodes, unless some type of constraint is imposed. Thus, the maximum areas allowed for ancestral nodes set to the minimum (two) to reduce ambiguities at the more basal nodes of the tree [119], [141], [142]. An exact optimization (versus heuristic) was invoked by allowing the maximum number of alternative reconstructions to be held at any node. The fifteen areas assigned to the 75 terminal taxa in our matrix were: A = South Africa, B = Madagascar, C = Amazonas (north, east, south), D = Chile, E = Eastern Brazil, F = Central Brazil, G = Andes, H = Central America, I = Mexico, J = West Indies, K = Southern Brazil, L = Northern South America, i.e., Caribbean coastal Venezuela and Colombia, French Guiana, Guyana, Surinam, M = Argentina-Paraguay-Uruguay, N = Colombia-Venezuela (*llanos* region), and O = western Amazonas. For *Cocos nucifera*, which is pantropical, we assigned only those areas of these fifteen where the species is currently found.

### Leaflet anatomical sections

Leaflets of *Allagopetra*, *Parajubaea* and *Polyandrococos* were hand sectioned with a double edged razor blade on a cutting board after folding the leaflet back and forth on itself various times to facilitate sectioning. Sections were not stained. Dried material was boiled for 5–10 minutes in water with Aerosol Detergent (Stepahn Co., Northfield, IL).

## Supporting Information

Figure S1 Strict consensus trees from parsimony analysis for loci WRKY2-12 (four loci).
(1.31 MB TIF)

Figure S2 Strict consensus trees from parsimony analysis for loci WRKY16, 19, and 21 (three loci).
(1.00 MB TIF)

Figure S3 Maximum likelihood bootstrap consensus trees for each of four WRKY loci: WRKY2, 6, 7, and 12.
(0.87 MB TIF)

Figure S4 Maximum likelihood bootstrap consensus trees for each of three WRKY loci: WRKY16, 19, and 21, as well as the combined analysis (all seven loci).
(0.94 MB TIF)

Figure S5 Strict consensus tree of 190 lowest “cost” reconciled species trees found by 25 heuristic searches by the program GeneTree using gene tree bootstrapping.
(0.08 MB TIF)

Table S1 All 75 taxa used in the study are listed with voucher specimens and GenBank accession numbers for the WRKY sequences.
(0.12 MB DOC)

Table S2 This table lists the specific primers used to amplify and sequence the seven WRKY loci from members of Arecaceaea tribe Cocoseae.
(0.03 MB DOC)
