# Correction: Phylogenetic Analysis of Seven WRKY Genes across the Palm Subtribe Attaleinae (Arecaceae) Identifies *Syagrus* as Sister Group of the Coconut

**DOI:** 10.1371/annotation/c59c150a-0412-490a-8e7d-ae5622fd4434

**Published:** 2009-10-23

**Authors:** Alan W. Meerow, Larry Noblick, James W. Borrone, Thomas L. P. Couvreur, Margarita Mauro-Herrera, William J. Hahn, David N. Kuhn, Kyoko Nakamura, Nora H. Oleas, Raymond J. Schnell

The word "Arecaceae" was misspelled in the article title. The correct title is: Phylogenetic Analysis of Seven WRKY Genes across the Palm Subtribe Attaleinae (Arecaceae) Identifies Syagrus as Sister Group of the Coconut. The correct citation is: Meerow AW, Noblick L, Borrone JW, Couvreur TLP, Mauro-Herrera M, et al. (2009) Phylogenetic Analysis of Seven WRKY Genes across the Palm Subtribe Attaleinae (Arecaceae) Identifies Syagrus as Sister Group of the Coconut. PLoS ONE 4(10): e7353. doi:10.1371/journal.pone.0007353 

