# Supplementary figures and images for: Phylogenetic Analysis of Seven WRKY Genes across the Palm Subtribe Attaleinae (Arecaceae) Identifies Syagrus as Sister Group of the Coconut
Source: PLoS One. 2009 Oct 6;4(10):e7353. doi: 10.1371/journal.pone.0007353 (PMC2752195; doi:10.1371/journal.pone.0007353)

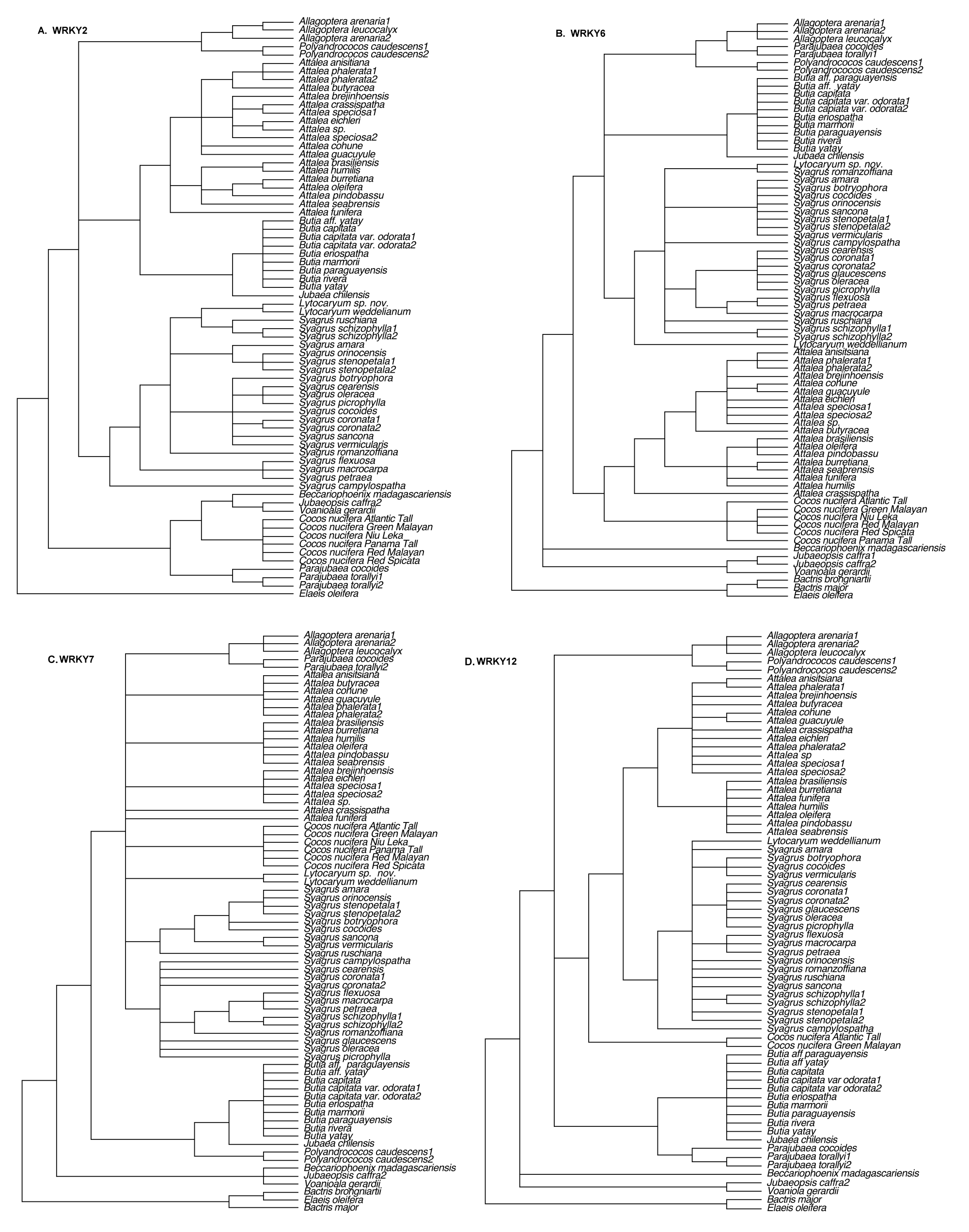

Supplement: Figure S1 — Strict consensus trees from parsimony analysis for loci WRKY2-12 (four loci). (1.31 MB TIF) [file pone.0007353.s001.tif]

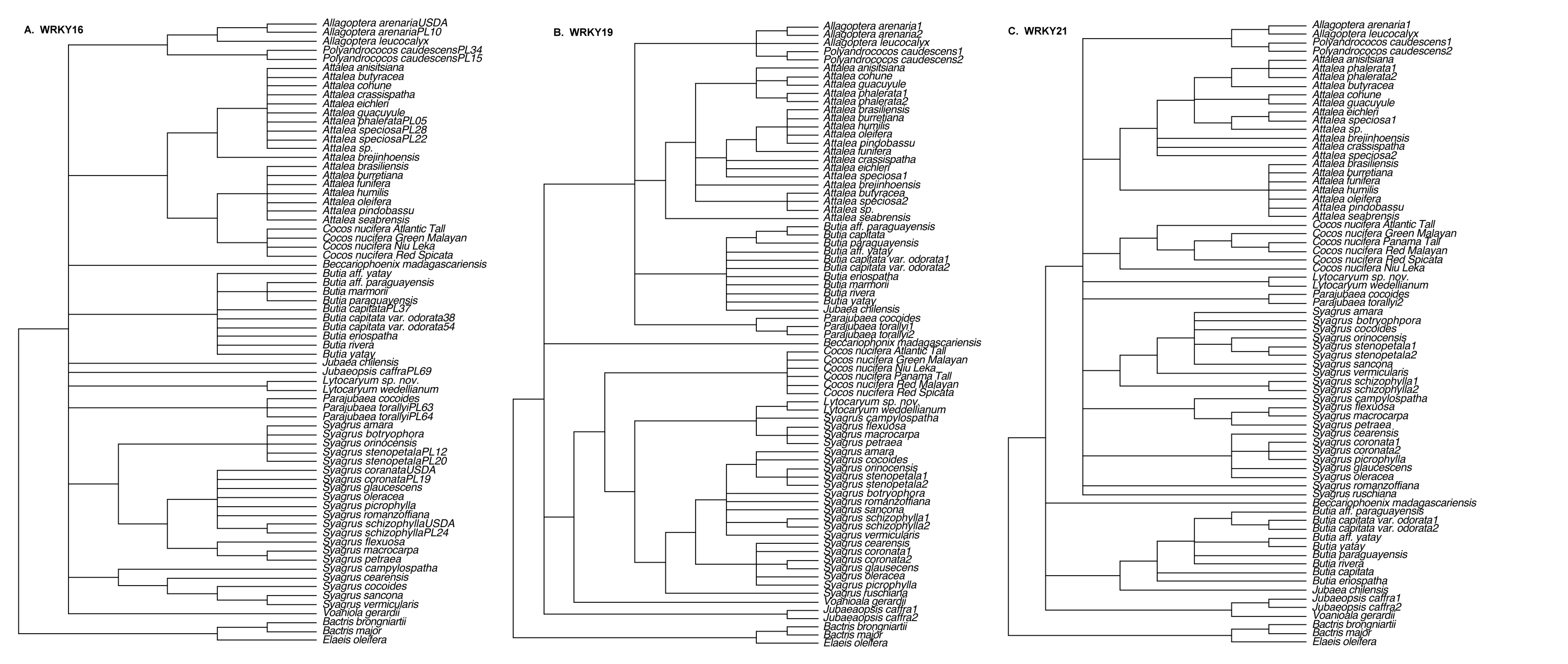

Supplement: Figure S2 — Strict consensus trees from parsimony analysis for loci WRKY16, 19, and 21 (three loci). (1.00 MB TIF) [file pone.0007353.s002.tif]

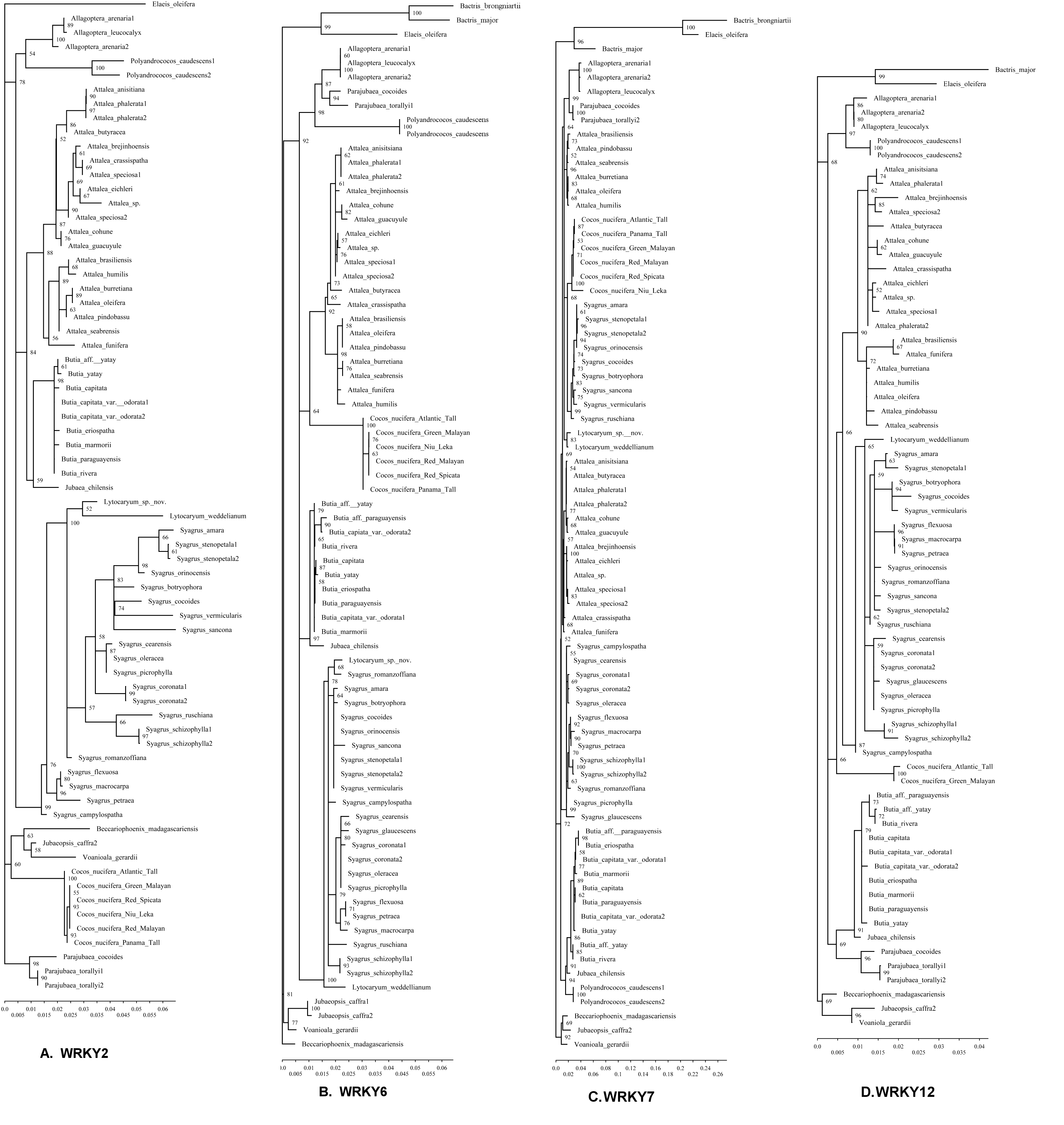

Supplement: Figure S3 — Maximum likelihood bootstrap consensus trees for each of four WRKY loci: WRKY2, 6, 7, and 12. (0.87 MB TIF) [file pone.0007353.s003.tif]

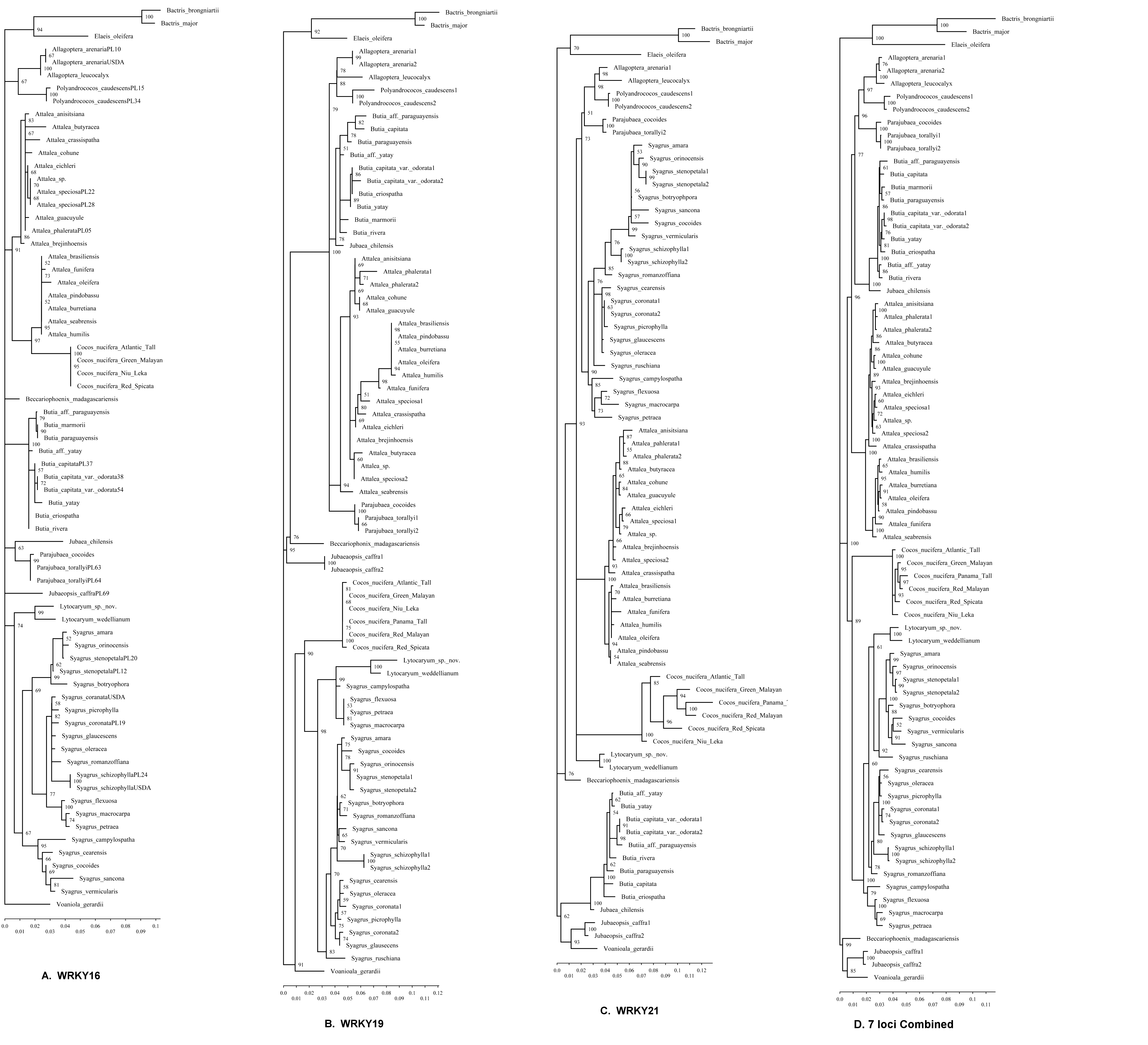

Supplement: Figure S4 — Maximum likelihood bootstrap consensus trees for each of three WRKY loci: WRKY16, 19, and 21, as well as the combined analysis (all seven loci). (0.94 MB TIF) [file pone.0007353.s004.tif]

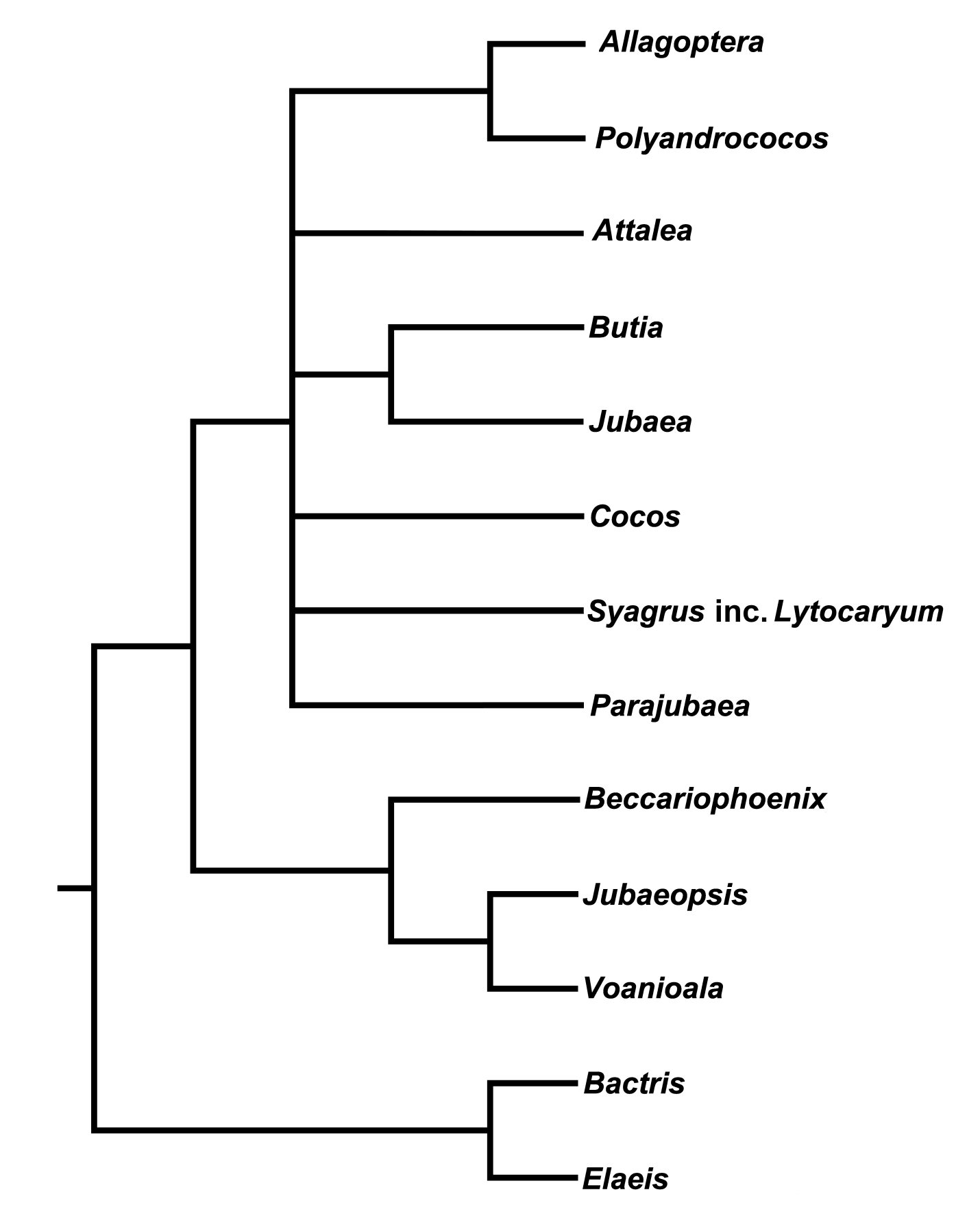

Supplement: Figure S5 — Strict consensus tree of 190 lowest “cost” reconciled species trees found by 25 heuristic searches by the program GeneTree using gene tree bootstrapping. (0.08 MB TIF) [file pone.0007353.s005.tif]
