# Supplementary material for: Phylogenetic Analysis of Seven WRKY Genes across the Palm Subtribe Attaleinae (Arecaceae) Identifies Syagrus as Sister Group of the Coconut
Source: PLoS One. 2009 Oct 6;4(10):e7353. doi: 10.1371/journal.pone.0007353 (PMC2752195; doi:10.1371/journal.pone.0007353)
Supplement: Table S1 — All 75 taxa used in the study are listed with voucher specimens and GenBank accession numbers for the WRKY sequences. (0.12 MB DOC) [file pone.0007353.s006.doc]

Table S1. Taxa, voucher specimens and GenBank sequence accession numbers of Arecaceae tribe Cocoseae used in the cladistic analysis of WRKY loci. All vouchers deposited at FTG unless otherwise indicated. An * preceding a GenBank number = PCR product had to be cloned before sequencing. nv = no voucher. -- = not successfully amplified.

| Species | Voucher |  |  | Locus | |  |  |  |
| --- | --- | --- | --- | --- | --- | --- | --- | --- |
|  |  | WRKY2 | WRKY6 | WRKY7 | WRKY12 | WRKY16 | WRKY19 | WRKY21 |
| *Allagoptera arenaria*1 (Gomes) Kuntze | Noblick 5168 (IPA) | FJ956927 | FJ9577069 | FJ957143 | FJ957216 | FJ957284 | FJ957354 | *FJ956997 |
| *A. arenaria*2 | Meerow 3200 (NA) | FJ956928 | FJ957070 | FJ957144 | FJ957217 | FJ957285 | FJ957355 | -- |
| *A. leucocalyx*  (Mart.) Kuntze | Zardini 54930 | FJ956929 | FJ957071 | FJ957145 | FJ957218 | FJ957286 | FJ957356 | FJ956998 |
| *Attalea anisitsiana*  (Barb. Rodr.) Zona | Noblick 5291 | FJ956930 | FJ957072 | FJ957146 | FJ957219 | FJ957287 | *FJ957357 | FJ956999 |
| *A. brasiliensis* Glassman | Noblick 5162 (IPA) | FJ956931 | FJ957073 | FJ957147 | FJ957220 | FJ957288 | FJ957358 | FJ9567000 |
| *A. brejinhoensis*  L. R. Noblick | Noblick 4614 | FJ956932 | *FJ957074 | FJ957148 | FJ957221 | *FJ957289 | *FJ957359 | FJ9567001 |
| *A. burretiana* Bondar | Noblick 4574 (BAH) | FJ956933 | FJ957075 | FJ957149 | FJ957222 | FJ957290 | FJ957360 | *FJ957002^†‡^ |
| *A. butyracea* (Mutis ex L. f.) Wess. Boer | Noblick 5513 | FJ956934 | FJ957076 | FJ957150 | FJ957223 | FJ957291 | FJ957361 | FJ957003 |
| *A. cohune* Mart. | Noblick 5519 | FJ956935 | FJ957077 | FJ957151 | FJ957224 | FJ957292 | *FJ957362 | FJ957004 |
| *A. crassispatha* (Mart.) Burret | Noblick 5516 | FJ956936 | FJ957078 | FJ957152 | FJ957225 | FJ957293 | FJ9573563 | FJ957005 |
| *A. eichleri* (Drude) Henderson | George Eiton 26 (nv) | FJ956937 | FJ957079 | FJ957153 | FJ957226 | FJ957294 | FJ957364 | FJ957006 |
| *A. funifera*  Mart. ex Spreng. | Noblick 5136 (IPA) | FJ956938 | *FJ957080 | FJ957154 | FJ957227 | FJ957295 | FJ957365 | FJ957007 |
| *A. guacuyule* (Liebm. ex Mart.) Zona | Noblick 4928 | FJ956939 | FJ957081 | FJ957155 | FJ957228 | FJ957296 | FJ957366 | *FJ957008^†‡^ |
| *A. humilis* Mart. ex Spreng. | Noblick 4575 (BAH) | FJ956940 | FJ957082 | FJ957156 | FJ957229 | FJ957297 | *FJ957367 | FJ957009 |
| *A. oleifera*  Barb. Rodr. | Noblick 5133 (IPA) | FJ956941 | FJ957083 | FJ957157 | FJ957230 | *FJ957298 | FJ957368 | *FJ957010^†‡^ |
| *A. phalerata*1 Mart. ex Spreng. | Noblick 5518 | FJ956942 | FJ957084 | FJ957158 | FJ957231 | FJ957299 | FJ957369 | FJ957011 |
| *A. phalerata*2 | Noblick 5018 | FJ956943 | FJ957085 | FJ957159 | FJ957232 | FJ957300 | FJ957370 | FJ957012 |
| *A. pindobass* Bondar | Noblick 5141 (IPA) | FJ956944 | FJ957086 | FJ957160 | FJ957233 | *FJ957301 | FJ957371 | FJ957013 |
| *A. seabrensis* Glassman | Noblick 4600 (BH) | FJ956945 | FJ957087 | FJ957161 | FJ957234 | FJ957302 | FJ957372 | *FJ957014^†‡^ |
| *A. speciosa*1 Mart. ex Spreng. | Noblick 4963 | FJ956946 | FJ957088 | FJ957162 | FJ957235 | FJ957303 | FJ957373 | FJ957015 |
| *A. speciosa*2 | Noblick 4950 | FJ956947 | FJ957089 | FJ957163 | FJ957236 | *FJ957304 | FJ957374 | FJ957016 |
| *A.* sp. | Noblick 5517 | FJ956948 | FJ957090 | FJ957164 | FJ957237 | FJ957305 | FJ957375 | FJ957017 |
| *Bactris brongniartii* Mart. | Noblick 4935 | -- | FJ957091 | *FJ957165^‡^ | -- | FJ957306 | FJ957376 | FJ957018 |
| *B. major* Jacq. | Noblick 4942 | -- | FJ957092 | *FJ957166^‡^ | FJ957238 | FJ957307 | FJ957377 | *FJ957019 |
| *Beccariophoenix madagascariensis* Jum. & H. Perrier | Noblick 5512 | FJ956949 | FJ957093 | FJ957167 | FJ957239 | FJ957308 | FJ957378 | FJ957020 |
| *Butia* aff. *paraguayensis* (Barb. Rod.) L. H. Bail. | Noblick 5459 | -- | FJ957094 | *FJ957168 | FJ957240 | *FJ957309 | FJ957379 | FJ957021 |
| *B.* aff. *yatay* (Mart.) Becc. | Elsa Zardini s.n. | FJ956950 | FJ957095 | FJ957169 | FJ957241 | FJ957310 | FJ957380 | *FJ957022^†‡^ |
| *B. capitata* (Mart.) Becc. | Noblick 5090 | FJ956951 | FJ957096 | FJ957170 | FJ957242 | FJ957311 | FJ957381 | FJ957023 |
| *B. capitata* var. *odorata*1 | Noblick 5130 | FJ956952 | FJ957097 | FJ957171 | FJ957243 | FJ957312 | FJ957382 | *FJ957024^†‡^ |
| *B. capitata* var. *odorata*2 | Noblick 5465 | *FJ956953^†^ | FJ957098 | FJ957172 | FJ957244 | *FJ957313^†^ | FJ957383 | *FJ957025^†‡^ |
| *B. eriospatha* (Mart.) Becc. | Noblick 4878 | FJ956954 | FJ957099 | FJ957173 | FJ957245 | FJ957314 | FJ957384 | *FJ957026^†‡^ |
| *B. lallemontii* L. R. Noblick | Noblick 5457 | FJ956957 | FJ957100 | FJ957174 | FJ957246 | FJ957315 | FJ957385 | FJ9570027 |
| *B. marmorii*  L. R. Noblick | Noblick 5336 | FJ956955 | FJ957101 | *FJ957175 | FJ957247 | FJ957316 | *FJ957386 | -- |
| *B. paraguayensis* | Noblick 5285 | FJ956956 | *FJ957102 | FJ957176 | FJ957248 | FJ957317 | FJ957387 | *FJ957028^†‡^ |
| *B. yatay* | Noblick 5461 | FJ956958 | FJ957103 | FJ957177 | FJ957249 | FJ957318 | FJ957388 | FJ957029 |
| *Cocos nucifera* L. 'Atlantic Tall' | Meerow 3201 (NA) | FJ956959 | FJ957104 | FJ957178 | FJ957250 | FJ957319 | FJ957389 | FJ957030 |
| *C*. *nucifera* 'Green Malayan Dwarf' | Meerow 3202 (NA) | FJ956960 | FJ957105 | FJ957179 | FJ957251 | -- | FJ957390 | FJ957031 |
| *C*. *nucifera* 'Niu Leka' | Meerow 3203 (NA) | FJ956961 | FJ957106 | FJ957180 | -- | FJ957320 | FJ957391 | FJ957032 |
| *C*. *nucifera* 'Panama Tall' | Meerow 3204 (NA) | FJ956962 | FJ957107 | FJ957181 | -- | -- | FJ957392 | FJ957033 |
| *C*. *nucifera* 'Red Malayan Dwarf' | Meerow 3205 (NA) | FJ956963 | FJ957108 | FJ957182 | -- | -- | FJ957393 | FJ957034 |
| *C*. *nucifera* 'Red Spicata' | Meerow 3206 (NA) | FJ956964 | FJ957109 | FJ957183 | -- | FJ957321 | FJ957394 | FJ957035 |
| *Elaeis oleifera* (Kunth) Cortés | Noblick 5521 | FJ956965 | FJ957110 | FJ957184^‡^ | FJ957252 | FJ957322 | FJ957395 | FJ957036 |
| *Jubaea chilensis* (Molina) Baill. | J.C.Pintaud 287 (MPU) | FJ956966 | FJ957111 | FJ957185 | FJ957253 | FJ957323 | FJ957396 | *FJ957037^†‡^ |
| *Jubaeopsis caffra*1 Becc. | J.C.Pintaud 279 (MPU) | -- | FJ957112 | -- | -- | -- | *FJ957397 | *FJ957038^†‡^ |
| *J. caffra*2 | HUNT (photo only) | *FJ956967^†^ | *FJ957113 | *FJ957186^‡^ | FJ957254 | * FJ957324 | *FJ957398 | *FJ957039 |
| *Lytocaryum weddellianum* (H. Wendl.) Tol. | Meerow 3207 (NA) | *FJ956968^†^ | FJ957114 | *FJ957187 | FJ957255 | FJ957325^†^ | *FJ957399 | *FJ957040 |
| *Lytocaryum* sp. nov. | Lorenzi 6496 | FJ956969 | FJ957115 | FJ957188^‡^ | FJ957256 | FJ957326 | FJ957400 | *FJ957041 |
| *Parajubaea cocoides* Burret | J. C. Pintaud 282 (MPU) | FJ956970 | FJ957116 | FJ957189 | FJ957257 | FJ957327 | FJ957401 | *FJ957042^†‡^ |
| *P. torallyi*1 (Mart.) Burret | Elva West 1 (nv) | FJ956971 | FJ957117 | -- | FJ957258 | FJ957328 | FJ957402 | *FJ957043 |
| *P. torallyi*2 | Elva West 2 (nv) | FJ956972 | -- | FJ957190 | FJ957259 | FJ957329 | FJ957403 | -- |
| *Polyandrococos caudescens*1 (Mart.) Barb. Rodr. | Noblick 5135 | FJ956973 | FJ957118 | FJ957191 | FJ957260 | FJ957330 | FJ957404 | FJ957044 |
| *P. caudescens*2 | Noblick 5003 (IPA) | FJ956974 | FJ957119 | FJ957192 | FJ957261 | *FJ957331 | FJ957405 | *FJ957045^†‡^ |
| *Syagrus amara* (Jacq.) Mart. | Hahn 7649 (NY) | FJ956975 | FJ957120 | FJ957193 | FJ957262 | FJ957332 | FJ957406 | *FJ957046 |
| *S. botryophora* (Mart.) Mart. | Noblick 5002 | FJ956976 | FJ957121 | FJ957194 | FJ957263 | FJ957333 | FJ957407 | FJ957047 |
| *S. campylospatha* (Barb. Rodr.) Becc. | Noblick 5128 | *FJ956977 | FJ957122 | FJ957195 | FJ957264 | *FJ957334 | FJ957408 | FJ957048 |
| *S. cearensis* L. R. Noblick | Noblick 5132 (IPA) | *FJ956978 | FJ957123 | FJ957196 | FJ957265 | FJ957335 | *FJ957409 | FJ957049 |
| *S. cocoides* Mart. | Noblick 4954 | FJ956979^†^ | FJ957124 | FJ957197 | FJ957266 | *FJ957336 | FJ957410 | FJ957050 |
| *S. coronata*1 (Mart.) Becc. | Noblick 4833 | FJ956980 | FJ957125 | FJ957198 | FJ957267 | FJ957337 | *FJ957411 | *FJ957051^†‡^ |
| *S. coronata*2 | Meerow 3208 (NA) | FJ956981 | FJ957126 | FJ957199 | FJ957268 | FJ957338 | *FJ957412 | *FJ957052^†‡^ |
| *S. flexuosa* (Mart.) Becc. | Noblick 5108 | -- | FJ957127 | FJ957200 | FJ957269 | *FJ957339 | FJ957413 | *FJ957053^†‡^ |
| *S. glaucescens* Glaz. Ex Becc. | Noblick 5511 | FJ956982^†^ | FJ957128 | FJ957201 | FJ957270 | FJ957340 | *FJ957414 | *FJ957054^†‡^ |
| *S. macrocarpa* Barb. Rodr. | Noblick 4841 | FJ956983 | FJ957129 | FJ957202 | -- | FJ957341 | *FJ957415 | *FJ957055 |
| *S. oleracea* (Mart.) Becc. | Noblick 5084 | *FJ956984 | FJ957130 | FJ957203 | FJ957271 | *FJ957342 | FJ957416 | *FJ957056^†‡^ |
| *S. orinocensis* (Spruce) Burret | Noblick 4946 | *FJ956985^†^ | FJ957131 | FJ957204 | FJ957272 | FJ957343 | FJ957417 | *FJ957057 |
| *S. petraea* (Mart.) Becc. | Noblick 5126 | *FJ956986^†^ | FJ957132 | FJ957205 | FJ957273 | *FJ957344 | FJ957418 | *FJ957058^†‡^ |
| *S. picrophylia* Barb. Rodr. | Noblick 5156 (IPA) | FJ956987^†^ | FJ957133 | FJ957206 | FJ957274 | *FJ957345 | FJ957409 | *FJ957059^†‡^ |
| *S. romanzoffiana* (Cham.) Glassman | Noblick 5167 (PY) | *FJ956988 | FJ957134 | FJ957207 | FJ957275 | *FJ957346 | FJ957420 | *FJ957060 |
| *S. ruschiana* (Bondar) Glassman | Noblick 5169 (IPA) | FJ956989^†^ | FJ957135 | FJ957208 | FJ957276 | -- | FJ957421 | FJ957061 |
| *S. sancona* H. Karst. | Noblick 5514 | FJ956990 | FJ957136 | FJ957209 | FJ957277 | FJ957347^†^ | FJ957422 | FJ957062 |
| *S. schizophylla*1 (Mart.) Glassman | Noblick 5134 (IPA) | FJ956991 | FJ957137 | FJ957210 | FJ957278 | FJ957348 | FJ957423 | FJ957063 |
| *S. schizophylla*2 | Meerow 3209 (NA) | FJ956992 | FJ957138 | FJ957211 | FJ957279 | FJ957349 | FJ957424 | *FJ957064^†‡^ |
| *S. stenopetala*1 Burret | Noblick 4936 | *FJ956993^†^ | FJ957139 | FJ957212 | FJ957280 | FJ957350 | FJ957425 | *FJ957065^†‡^ |
| *S. stenopetala*2 | Noblick 5515 | FJ956994 | FJ957140 | FJ957213 | FJ957281 | FJ957351 | FJ957426 | *FJ957066 |
| *S. vermicularis* L. R. Noblick | Noblick 4974 | FJ956995 | FJ957141 | FJ957214 | FJ957282 | FJ957352 | FJ957427 | FJ957067 |
| *Voanioala gerardii* J. Dransf. | Martin Sloos (nv) ^§^ | FJ956995 | FJ957142 | FJ957215 | FJ957283 | FJ957353^†^ | *FJ957428 | *FJ957068^†‡^ |

^†^Reverse primer # 2 (see S9), ^‡^forward primer # 2 (see S9), ^§^Moet Palm Research Center, Netherlands,
