# Supplementary material for: Phylogenetic Analysis of Seven WRKY Genes across the Palm Subtribe Attaleinae (Arecaceae) Identifies Syagrus as Sister Group of the Coconut
Source: PLoS One. 2009 Oct 6;4(10):e7353. doi: 10.1371/journal.pone.0007353 (PMC2752195; doi:10.1371/journal.pone.0007353)
Supplement: Table S2 — This table lists the specific primers used to amplify and sequence the seven WRKY loci from members of Arecaceaea tribe Cocoseae. (0.03 MB DOC) [file pone.0007353.s007.doc]

Table S2. WRKY primer sequences (5' 🡪 3').

| Locus | Forward | Reverse |
| --- | --- | --- |
| WRKY2 | ACAATCACCCCAGGCTTTCTCA | 1) ATTCCACAACCCCGATGCTTC  2) TGAGATCATGAGATGCCCTCTCAA |
| WRKY6 | CCAAACCCAAGGTAGGTTTCAGC | CCTAACAGGGCACCCAGCATT |
| WRKY7 | 1) ACCCAAAGCCTCCACACA  2) GGCAGATCACTGAGTTGTGT | TCACCGCCCTTGGATCAT |
| WRKY12 | GGGTGCTCACAACCACTCCA | TGCCCTCTCCACATGCTTTC |
| WRKY16 | AGCCGTCAAAAACAGCCCATT | 1) CAAAGCAGCCACCGAGTTACA  2) GGAGGATCGCTCGACTCGACTCGTTTT |
| WRKY19 | AACAGCCCAAATCCAAGGTAT | TCCACTCTCTTCTTTACAGAACAAC |
| WRKY21 | 1) AGAGTAACCCATGCCCACGA  2) ACCAGCATGCCCGGTGAA | 1) GCACACCTCTGCACCTGAAA  2) ATGGACATGTCTTCAGCACACCTC |
